# Identification of Yeast Transcriptional Regulation Networks Using Multivariate Random Forests

**DOI:** 10.1371/journal.pcbi.1000414

**Published:** 2009-06-19

**Authors:** Yuanyuan Xiao, Mark R. Segal

**Affiliations:** Department of Epidemiology and Biostatistics, Center for Bioinformatics and Molecular Biostatistics, University of California, San Francisco, California, United States of America; Carnegie Mellon University, United States of America

## Abstract

The recent availability of whole-genome scale data sets that investigate complementary and diverse aspects of transcriptional regulation has spawned an increased need for new and effective computational approaches to analyze and integrate these large scale assays. Here, we propose a novel algorithm, based on random forest methodology, to relate gene expression (as derived from expression microarrays) to sequence features residing in gene promoters (as derived from DNA motif data) and transcription factor binding to gene promoters (as derived from tiling microarrays). We extend the random forest approach to model a multivariate response as represented, for example, by time-course gene expression measures. An analysis of the multivariate random forest output reveals complex regulatory networks, which consist of cohesive, condition-dependent regulatory cliques. Each regulatory clique features homogeneous gene expression profiles and common motifs or synergistic motif groups. We apply our method to several yeast physiological processes: cell cycle, sporulation, and various stress conditions. Our technique displays excellent performance with regard to identifying known regulatory motifs, including high order interactions. In addition, we present evidence of the existence of an alternative MCB-binding pathway, which we confirm using data from two independent cell cycle studies and two other physioloigical processes. Finally, we have uncovered elaborate transcription regulation refinement mechanisms involving PAC and mRRPE motifs that govern essential rRNA processing. These include intriguing instances of differing motif dosages and differing combinatorial motif control that promote regulatory specificity in rRNA metabolism under differing physiological processes.

## Author Summary

Transcriptional regulation, one of the most complex and intriguing processes in living cells, drives essential downstream cellular processes such as development, proliferation and differentiation. It gives rise to the versatility and flexibility that allows cells to determine their actions and states in response to internal needs or external stimuli by turning on, or shutting off, select sets of genes. This elaborate control of gene expression is realized by sophisticated transcriptional regulatory networks that include a diverse repertoire of transcription factors. Here, we study the relationship between gene expression and transcription factor binding in diverse yeast physiological processes. Our random forest-based method effectively models gene expression measurements simultaneously, bypassing the necessity of analyzing the multiple samples separately. Using our method, we have identified many high-order interactions between regulatory sequences that give rise to condition-specific gene expression.

## Introduction

Eukaryotic gene regulation is governed at many levels. At the transcriptional level, transcription factor (TF) binding, chromatin structure changes and multiple activators cooperate to promote an intricate and complex gene expression network. With the advance of high-throuput technologies, whole-genome scale data sets that investigate diverse aspects of transcription regulation are available. Whole genome sequences elucidate DNA elements in the promoter regions of genes, chromatin immunoprecipitation (ChIP) technologies coupled with tiling microarrays reveal transcription factor binding sites, and expression micorarrays provide whole genome expression profiles in response to genetic or environmental changes. New and effective bioinformatic tools are needed to integrate these large scale assays that provide complementary information on different levels of the regulatory process.

There have been many studies, and corresponding analytic approaches, that aim to address the challenge of eliciting regulatory networks using both regulatory element information and microarray expression data. Here following Boorsma *et al.* [1], we define regulatory elements as *regulons*, which can be motif counts or ChIP-based TF binding information. A widely-applied strategy involves first grouping genes with similar expression profiles using some clustering algorithm, such as hierarchical clustering. Then, a motif-finding algorithm is applied within each expression cluster to identify enriched sequence motifs in the promoters of its (gene) members [2]–[4]. These DNA sequence elements/motifs are assumed to act as binding sites for transcription regulation. Despite some success, this cluster-first approach has several drawbacks: (i) genes with correlated expression profiles might not be co-regulated by a common motif, (ii) genes with the motif might not respond, (iii) results are highly dependent on the clustering algorithm employed, and (iv) by prioritizing highly cohesive co-expression, it lacks the sensitivity to reveal subtle changes promoted by combinatorial regulation control. An improved and more sophisticated clustering approach that has the ability to incorporate both expression and regulatory information to define clusters is provided by the biclustering algorithm [5].

Motivated by benefits of directly modeling the regulon-expression relationship, and averting the shortcomings of cluster-first approaches, a number of subsequent strategies adopt formulations whereby regulons (motifs and/or ChIP-based TF binding information) constitute predictors, and expression measures outcomes. This suggests application of regression flavored techniques. Notable methods in this category include simple linear regression [6], logic regression [7], an iterative approach of clustering followed by regression tree where refinement of cluster membership and tree parametrization is aided by the EM algorithm [8], multivariate adaptive regression splines [9], multivariate regression trees [10], input-output hidden markov model [11], boosting [12] and projection-based approaches [13]. Utilizing a regression framework allows formal evaluation of both main- and interaction-effect contributions from motifs to expression levels. Interaction effects can be interpreted as motif cooperativity. Except for logic regression [7] and tree-based methods [10],[12], the above techniques assume additive contributions from motifs. Moreover, interaction effects can only be included when both constituent main effects are present due to the hierarchical nature of the models. In addition, most of these approaches are limited to handling one expression sample/response at a time, this being the case for the bulk of the regression methods surveyed in a recent review published in this journal [14]. Again, multivariate regression trees are an exception.

Indeed, the tree-based regression paradigm [15],[16] has many advantages for modeling regulon-expression relationships: (i) flexible extraction of important and/or interacting covariates (motifs, TF-bindings) among a large number thereof, (ii) no imposition of rigid parametric assumptions, either with regard (error) distributions or model functional form and, most importantly, (iii) it is formulated to identify (gene) subgroups with common covariate (motif, TF binding) values and homogeneous multiple outcomes (coherent expression profiles), simultaneously effecting regression and clustering analyses. Due to these desirable underpinings, we base our method on the multivariate regression tree (MRT) approach of Segal [17] and Phuong *et al.* [10]. MRT is a natural extension of the standard regression tree schema [15], in which a univariate response is replaced by a multivariate one, here the expression levels across multiple experimental conditions.

However, tree methods are not without their deficiencies [18], notably instability, modest prediction performance, and greediness in choosing splits. In the present application, where our focus is on identifying regulatory networks (interactions), the last shortcoming is arguably the most significant. Fortunately, in the context of univariate outcomes, these shortcomings have been remedied via the use of ensembles of individual trees, known as random forests [19]. Extensive benchmarking studies have shown that random forests enjoy improved prediction performance and minimized parameter tuning over single trees. Furthermore, by injecting randomness through both bootstrapping (each bootstrap replicate generating one member of the ensemble) and splitting on a random selection of covariates at each node, random forests effectively examine a large number splits and interactions, thereby yielding a much more complete catalog of important networks than a single tree. Here, we expand the scope of random forests to include multivariate outcomes. This is accomplished by generating an ensemble of MRTs. Accordingly, we designate our approach *Multivariate Random Forests* (MRF).

One important component of random forests output is the proximity matrix which can serve as a natural similarity metric that quantifies similitude based on *both* homogeneity in outcome (expression level) and covariates (motif counts and TF-bindings). We exploit this property of the proximity matrix and use it as a similarity matrix to conduct a “guided” clustering based on the PAM algorithm (Partition Around Medoid; [20]) to identify small cohesive *Regulatory Cliques* (RCs). Each RC contains genes that are co-expressed and co-regulated and can be described by its signature motifs and TF binding that are commonly present in its gene members. The derivation of these RCs based on information provided by the proximity matrix is a bottom-up approach that seeks to decipher the mechanics of random forests, often construed as a “black box”, by reconstructing and re-associating its inventory of effective splits (motifs and TF binding) with the resulting homogeneous nodes.

For predictor inputs, we used both motif counts and ChIP-based binding data for over 200 TFs performed in rich medium (YPD) by Harbison *et al.* [21]. For outcomes, we modeled expression data from the cell cycle [3],[22], sporulation [23] and various stress conditions: heat shock, nitrogen depletion, DTT exposure, and steady-state growth on alternative carbon sources [24]. We provide a rigorous assessment of MRF's utility in uncovering yeast regulatory networks. We utilize yeast cell cycle data [22] to illustrate the performance of MRF in elucidating both cyclic and non-cyclic RCs in the cell cycle, and compare findings not only to a suite of general statistical approaches, such as single multivariate regression trees, cluster analysis, and univariate random forests, but also to current computational methods, specifically devised to model yeast gene regulation [11],[13]. In addition, to further validate the stability and reproducibility of MRF's performance, we perform two additional comparisons, also based on the yeast cell cycle: (i) we compare findings of MRF using only motifs as predictors to those using both motifs and TF binding as predictors [22]; and (ii) we compare MRF's findings on two independent cell cycle data sets[3],[22]. Next, we examine yeast sporulation and a diverse set of stress conditions, and show that MRF can not only identify regulatory modules that are constitutively present across these different conditions, but also those that give rise to condition-specific responses to different environmental stimuli. Specifically, we provide evidence of the existence of an alternative MCB motif binding pathway. In addition, we outline an elaborate yeast transcription regulation refinement mechanism involving the PAC and RRPE motifs, effected by motif doses and combinations.

## Results

### MRF Implementation and Evaluation

**Figure 1:**
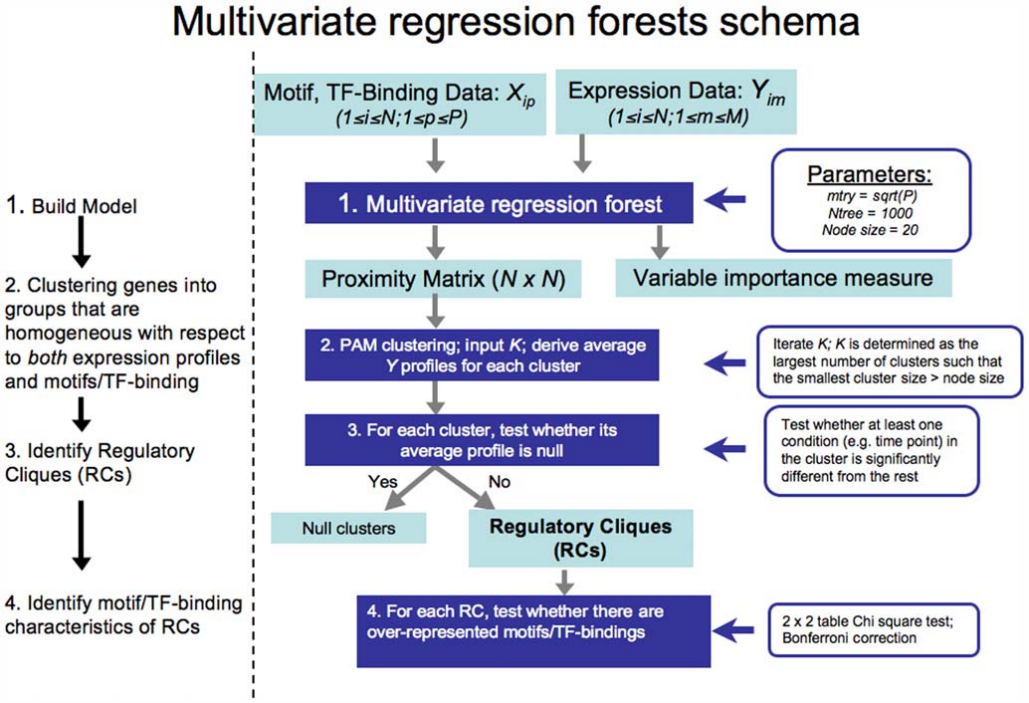
Schema of Multivariate Regression Forests.

The four steps of our MRF technique for identifying regulatory networks are illustrated in the schematic in Figure 1. Details are provided in Materials and Methods. Briefly, in the first step, we build a random forest comprising a large number of multivariate regression trees using motif and/or TF-binding data as predictors and expression data as outcomes. One useful MRF byproduct is a variable importance measure, which we use to assess a regulon's overall regulatory influence on gene expression, and for which we propose a randomization procedure to assess significance. The MRF also yields a proximity matrix that quantifies gene-gene similarity, based on both regulon and expression, which we use to decipher regulon-regulon interactions and regulon-expression associations. To this end, in the ensuing steps, we employ the PAM algorithm on the proximity matrix to allocate genes into homogeneous groups (step 2), and subsequently identify regulatory cliques (RCs) with tight, cohesive, and time-dependent profiles and the associated characterizing regulatory elements (steps 3 and 4).

#### A. MRF tree representation, prediction errors and variable importance output

**Figure 2:**
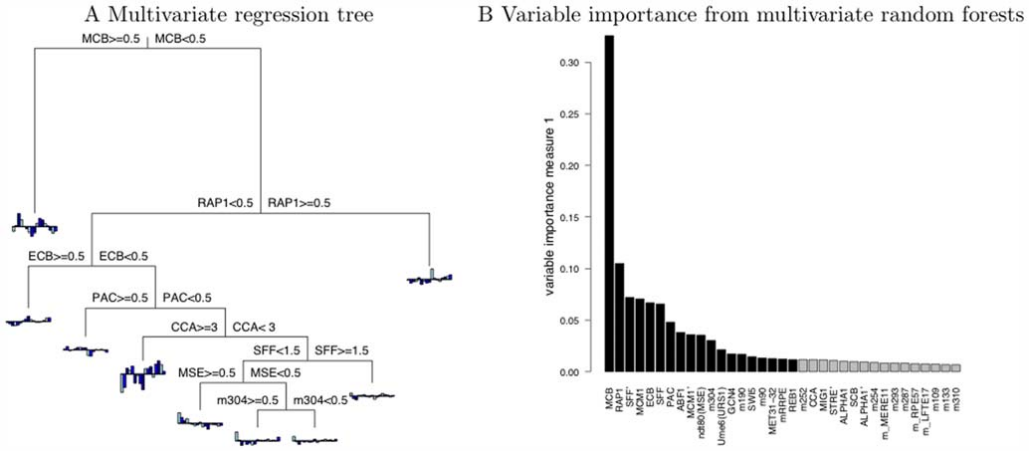
A Multivariate regression tree and the variable importance measures from MRF for the cell cycle data. Panel A illustrates a multivariate regression tree built for the cell cycle data, in which allowable splits are order-preserving motif counts and the splitting values are mid points of two consecutive motif counts. The first split separates MCB counts at 0.5, resulting in genes that have at least one copy of the MCB motif (≥0.5) going into the left daughter node whereas genes that don not have the MCB motif (<0.5) going into the right daughter node. Tree is pruned to the size that has the lowest cross validation error. At each leaf node resides a barplot that indicates the average expression level at each time point of genes allocated to the node. Panel B shows the barplot of the variable importance measures yielded by multivariate random forests (MRF). Black bars are those that have significant (FDR adjusted 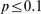) based on the permutation procedure that randomizes the relationship between expression and motif counts. Names of putative motifs begin with the letter “m”.

The multivariate regression tree for the cell cycle data using motif counts as predictors, with size determined by cross-validation, and built using the R package *mvpart* is shown in Figure 2A.We also illustrate a few exemplary multivariate trees built with bootstrapped samples of the original data and pruned to similar size in Text S1 Figure 1(A–C). Evident from this small selection of trees is the variability in tree topology and splits, underscoring the instability of single trees. Also apparent is the dominance of the MCB motif, which potentially precludes other motifs from emerging.

As indicated, random forests overcome these concerns by appropriately injecting randomness: each split within a constituent tree, from the bootstrap ensemble, is chosen from a sub-sample of the motifs. This provides opportunities for other candidate motifs and their attendant interactions to be examined, yielding a more diverse catalog of effective motifs and motifs combinations. The cross-validated relative prediction errors of the multivariate tree and the out-of-bag relative prediction errors of random forests for the cell cycle data are presented in Text S1 Figure 2. The lowest cross-validated relative prediction error for the tree is 97.1% and this is reduced to 95.3% for the forests using the cell cycle data. A similar scale of prediction error and error reduction is also observed in the other five array data sets that we investigate in this study (results not shown). We note that despite a significant decrease in prediction error compared to a single tree, the prediction power for forests is still meager due to (i) large between-gene variation, (ii) minimal pre-filtering of null genes, and (iii) the contributions of numerous other (unmeasured) factors, beyond motif counts, to expression levels.

The random forest algorithm outputs covariate importance summaries, which have been shown to be adept at identifying predictors that exert influences either by themselves or cooperatively with other predictors in high-dimensional genomics settings [25]–[29]. We plotted ordered importance measures from motifs that received significantly higher values (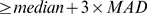) in Figure 2B. Among those identified from the 356 motifs are several experimentally verified motifs associated with the cell cycle: MCB, ECB, SCB, SFF' and MCM1'.

#### B. Method validation via randomization

**Figure 3:**
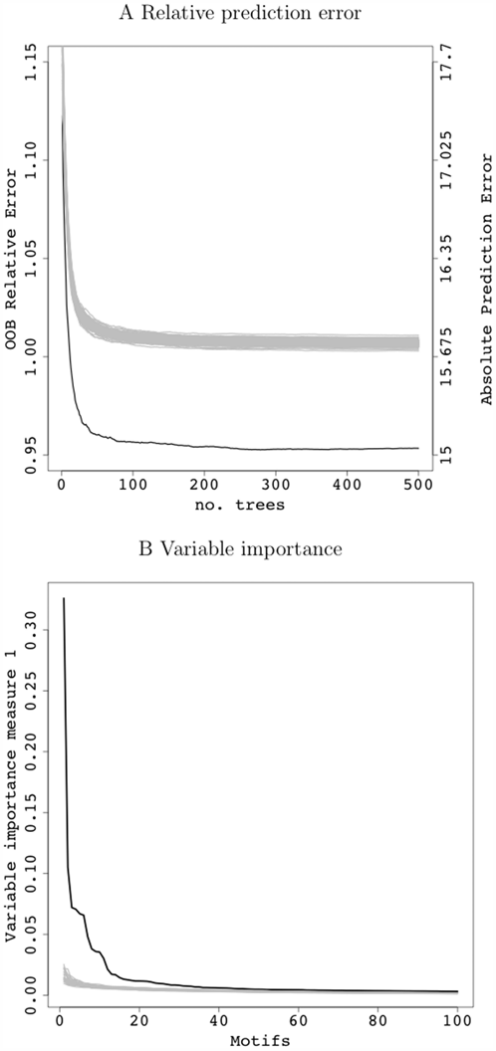
Outputs of (A) relative prediction error (left axis) and absolute prediction error (right axis) and (B) variable importance measures from MRT. Black traces are the real, observed statistics, whereas gray traces are derived from the 100 permuted data. Only top 100 ordered motifs are drawn in B.

An obvious question is whether the observed improved prediction performance and the highly ranked motifs (via variable importance measures) result from meaningful regulatory relationships. In the absence of experimental validation, we address this by disrupting the original motif (X matrix) – expression (Y matrix for the cell cycle data) correspondence by randomly permuting the rows of the expression matrix. So doing disassociates response-predictor relationships, but preserves within-predictor and within-response correlation structures. The relative prediction error traces and the ordered variable importance measures for the 100 permuted data sets (in gray) are displayed in contrast to those calculated from the original data set (in black) in Figure 3. The randomization process provides a means to assess model quality and significance of the observed summaries including relative prediction error and motif importance measures. This is carried out by computing the relative prediction error and motif importance measures for each permuted data set. A histogram is then formed for each statistic and a permutation *p* value derived. The permutation *p* values for variable importance were evaluated collectively and adjusted using the false discovery rate (FDR) control procedure proposed by Benjamini and Hochberg [30]. There are 19 motifs that have a FDR *p* value≤0.1, and they are highlighted in Figure 2B. A detailed discussion of motif importances and regulatory cliques for the cell cycle data follows.

### Application of MRF on Cell Cycle Using Motif Data as Predictors

The variable importance measures yielded by the random forests evaluate the contribution of each candidate motif to gene expression, but do not reveal whether this contribution is the result of individual-motif potency or multiple-motif synergies, nor do they disclose the constituent genes that the motif governs. One modest step with regard the question of synergy was recently proposed [26] but is limited to yielding pairwise importances: again combinatorial explosion precludes extending this approach. Since regression trees share similar attributes with clustering in partitioning genes into homogeneous groups, we pursue deciphering the mechanisms underlying co-expression and co-regulation, as modeled by random forests, by recovering these homogeneous gene groups, which we term regulatory cliques (RCs).

#### A. Cyclic RCs of the cell cycle

**Figure 4:**
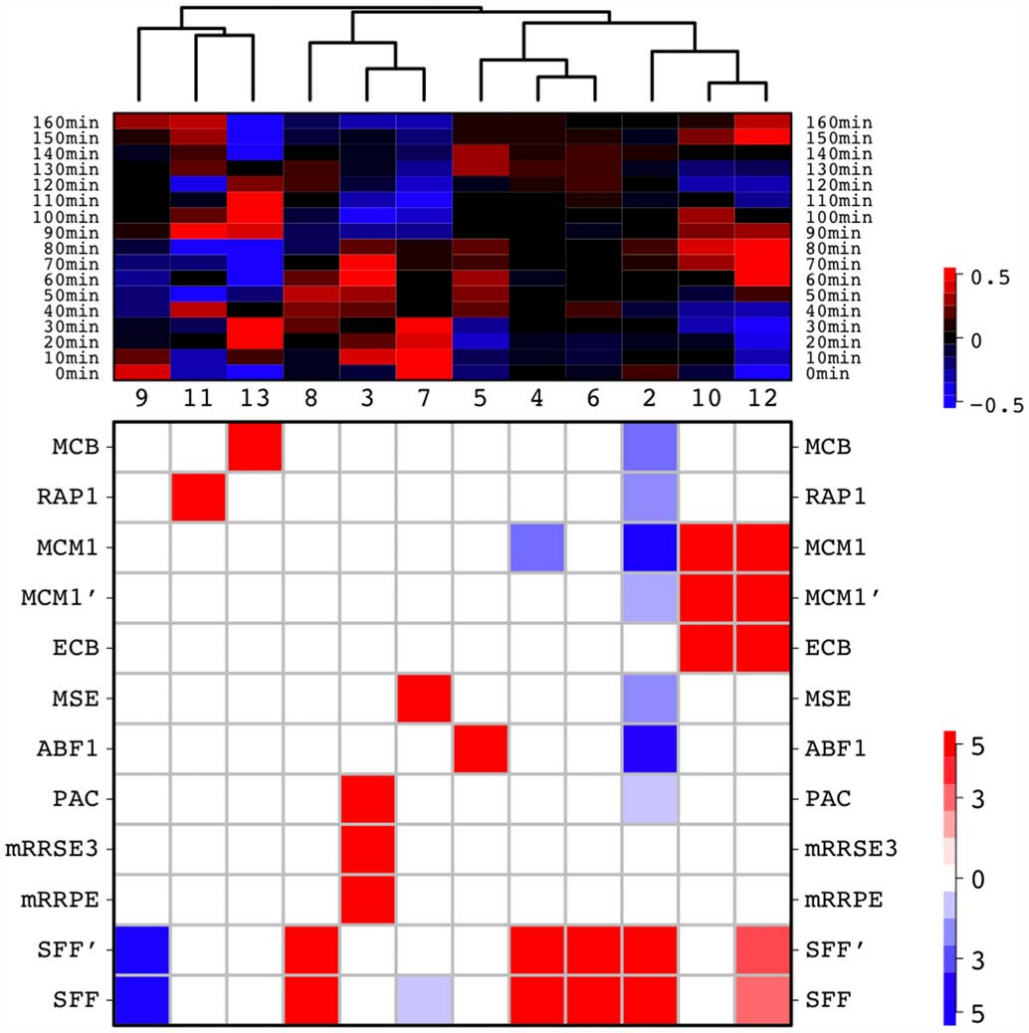
RC diagram of the cell cycle data by Cho *et al.* using motifs as predictors. Each column is an RC with the original cluster numbers designated by PAM indicated in the middle. Note cluster 1, which contains 599 genes, is designated as a null cluster and not shown. The top section of the graph shows the average expression profile of the genes in a specific RC, which is clustered based on Pearson correlation and average linkage. The magnitude of the expression in log_2_-ratios can be read off from the color bar at the top right hand side. The bottom section depicts signature motifs in the corresponding RC. The color red indicates enrichment 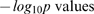 by a Chi-square test of association; the color blue corresponds to the depletion 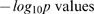. The color bar at the lower right hand side is in 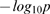 scale and the color signals the direction of the test.

**Figure 5:**
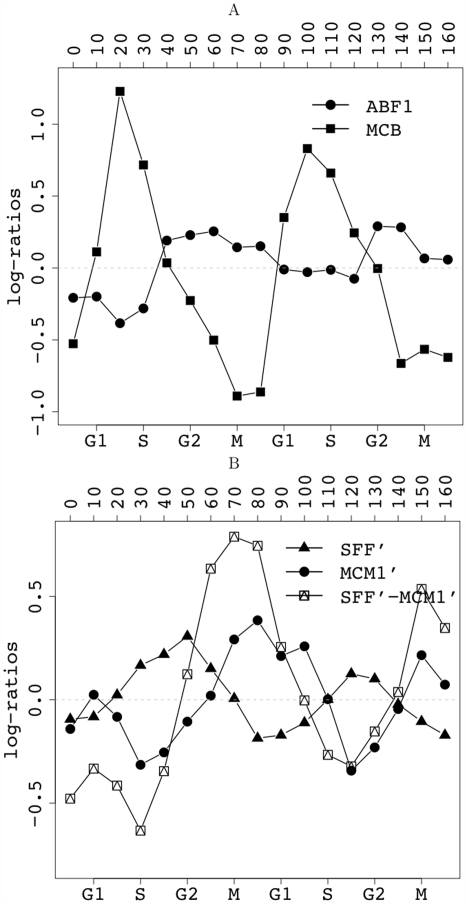
Time courses for (A) MCB and ABF1 RCs and (B) SFF' and MCM1' RCs in cell cycle. Plotted are traces of average expression profiles of the genes in the corresponding RC.

Derivation of RCs using PAM clustering on the proximity matrix and motif enrichment analysis (see Materials and Methods for details) gives rise to the RC diagram in Figure 4 for the cell cycle data set. Each column in the figure corresponds to an identified RC, with the upper panel depicting its average expression profile, and the lower panel highlighting its highly enriched / depleted motifs. One cluster, that contains 599 genes, is designated as null (step 3 in Figure 1) and its expression profile is not shown. These RCs can be divided into cyclic and non-cyclic expression patterns. The cyclic RCs include a total of 636 genes, the expression of which show large wave-like fluctuations, and can be divided into five cliques according to the time of their peak expression and their signature motifs: these are illustrated in Figures 4 and 5. The five cyclic RCs govern four different cell cycle phases. In the G1→S phase, when the initiation of DNA replication occurs, we identified MCB (*Mlu*I cell cycle box) as the single motif, whose target genes show strong wave-like expression pattern that peaks during the G1→S transition (Figure 5A). The MCB motif is the binding site of Mbp1, a transcription factor known to be involved in mitotic transcription from G1 to S phase [31]. In contrast, the ABF1 RC assumes a much weaker periodic expression pattern that peaks approximately at the G2 phase (Figure 5A). The ABF1 motif is the binding site of the general regulatory factor Abf1, whose contribution to mitotic promoter activities was previously confirmed via mutational analysis of its DNA-binding and protein-interaction domains [32].

In addition to these two motifs, our algorithm has also recovered three RCs of SFF', MCM1' and their combination SFF'-MCM1' (Figure 5B). Note that the SFF' motif is a subsequence of SFF. We do not adopt the approach of Pilpel *et al.* [33] of considering SFF and SFF' as synergistic when they appear in the same clique. Rather, we employ a more cautious approach, using the smaller SFF' motif to represent both motifs. Similarly, MCM1, MCM1' and ECB are variants of each other, and we use the shortest motif MCM1' to represent occurrences of all three motifs. SFF' (Swi five factor) is recognized by the conserved forkhead family of transcription factors Fkh1p and Fkh2p. The involvement of the Fkh proteins together with Mcm1p (MADS-box protein, recognizes the MCM1' motif) to regulate transcription of genes during the G2/M transition has been well established [34]. However, here we provide evidence that there is a set of 278 genes that may be regulated by Fkh proteins at the G2 phase independently of Mcm1p. The MCM1' RC contains a set of 122 genes, characterized by the MCM1' motif and a periodic expression pattern peaking during mitotic exit (M→G1; [35],[36]). The third RC in Figure 5B involves a set of genes that contain both of the SFF' and the MCM1' motifs. These genes are expressed at intermediate times (M phase) between the two transcriptional waves promoted by each individual regulator (G2 for SFF' or M→G1 for MCM1'). This is consistent with other findings [35],[37] that two cooperating/synergistic regulators can govern gene expression through at least three waves of gene expression, contributing to the refinement and sophistication of cell cycle transcription regulation.

#### B. Dosage effect of the SFF' motif in the cell cycle

**Figure 6:**
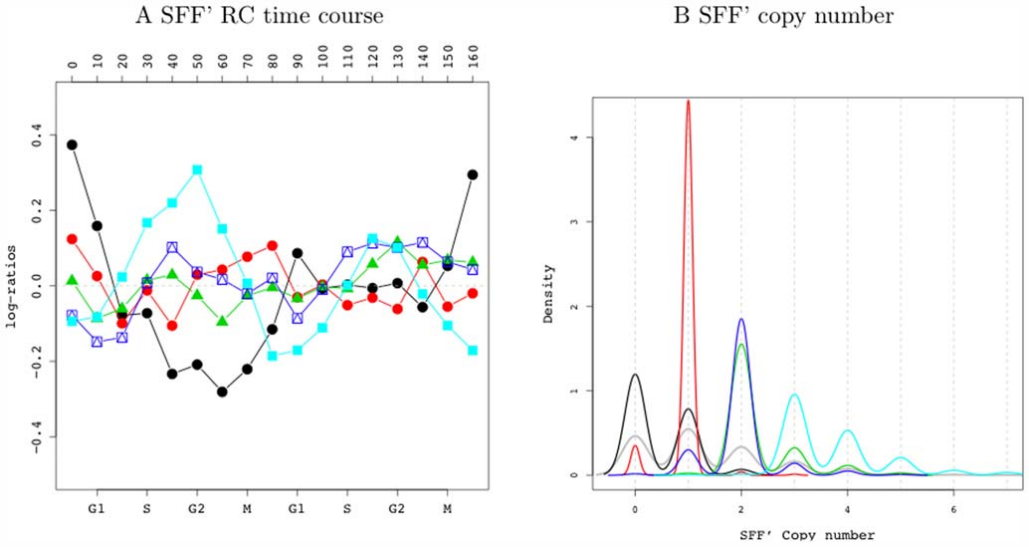
Time courses and dosages of SFF' for the multiple SFF' RCs in cell cycle. Panel A depicts SFF' RC's time profiles with different RCs differentiated by the different colors and their corresponding SFF' dosages are illustrated in B.

Another particularly notable feature of the SFF' RC is that almost all of these 278 gene targets have a high dosage of SFF' binding sites. In fact, 98.9% have at least 3 SFF' sites, whereas the overall frequency (all genes) is 17.1%. Similarly, 46.4% have at least 4 SFF' sites whereas the overall frequency is 7%. This finding led us to investigate whether there is a dosage effect of the SFF' sites on mitotic gene expression. We pursued this by examining the expression profiles of all RCs that involve the SFF' motifs. Mean gene expression profiles of these RCs are plotted in Figure 6A and the corresponding SFF' copy number distributions are illustrated in Figure 6B. RCs for genes containing 2 binding sites of SFF' (green and blue traces) show slight periodic expression patterns, with the periodicity magnified with increasing SFF' copies (cyan trace). This provides evidence that the strength of Fkh proteins in regulating cell cycle gene expression is directly proportional to the dosage of its binding sites. Intriguingly, a lack of SFF' sites (black trace) inverts the expression profile, with genes in this group having decreased expression at the G2 phase, as opposed to increased expression at this phase for genes with at least two SFF' sites.

#### C. Non-cyclic RCs of the cell cycle

In addition to the cyclic RCs, we also found several non-cyclic RCs, which contain genes whose expression is altered during the cell cycle experiment but not in any particular oscillatory pattern. These include cliques of RAP1, PAC-mRRPE-mRRPE3 and MSE. The mRRPE (also known as M3a) motif is derived from the MIPS rRNA-processing functional category, and PAC (also known as M3b) is found upstream of many DNA polymerase A and C genes. Both mRRPE and PAC have been identified to be enriched in the same expression cluster in previous analyses of the cell-cycle data [4],[33]. However, we also found that mRRSE3 (MIPS rRNA Synthesis Element 3) is highly enriched in the same clique. The discovery of this three-member regulatory module was the focus of Pilpel *et al.* [38], which deduced tri-membership through examining all pairwise synergies. The comparative ease with which our MRF algorithm identified this three-member RC demonstrates its effectiveness in eliciting high-order interactions. The RAP1 motif has a pivotal role in activating the transcription of ribosomal proteins, and the MSE motif is highly involved in meiosis gene regulation; our findings on the role of these motifs in the cell cycle are consistent with previous research [6],[33].

#### D. Comparisons of MRF to univariate RFs

In the last section, using motif and cell cycle expression data, we compared multivariate random forests to multivariate trees and showed that the forest ensemble improved prediction error and was more comprehensive in uncovering interactions (networks). We next investigate whether there are benefits to simultaneously modeling multiple responses, as opposed to making recourse to existing methods only equipped to handle univariate responses. First, we use (univariate) random forests [19], constructing the univariate response from the expression matrix via principal component analysis (PCA). PCA is a dimension reduction algorithm that replaces the original variables with orthogonal (uncorrelated) linear combinations thereof. The first principal component explains the maximal amount of outcome (expression) variability, and so on in decreasing order. In lieu of MRF, we reduced to a single outcome as provided by the first principal component (which explains 23% of the overall variance) and then applied random forests and PAM on the resulting proximity matrix as described in the method section. The derived RCs displayed in Text S1 Figure 3 include MCB, ABF1, MSE, RAP1, mRRPE-PAC-mRRSE3 and MCM1', a subset of what emerged when modeling the multivariate response. This highlights the robustness of the guided clustering component of the algorithm and its ability to recover relevant RCs. Nonetheless, this reduction of the multivariate outcome to a single principal component entails information loss, reflected in the absence of key RCs, including SFF' and SFF'-MCM'.

Next, we obtained a series of univariate random forests, each based on a specific time point. This can be a tedious process when many time points are involved and when gene expression at individual time points is not of primary interest, with synthesis of results across the respective model outputs being challenging. In a similar, time point specific approach of Bussemaker *et al.* [6], assimilation across models was achieved by graphing (main effect) regression coefficients, for a few prominent motifs, against time point. Correspondingly, we list the top 10 motifs at each time point by the random forests importance measure in Text S1 Table 1, in addition to plotting normalized importance measure traces for the three motifs that are ranked the most important for at least one of the 16 time points – MCB, SFF and RAP1 – in Text S1 Figure 4. It is evident from these results that even though modeling each time point separately reveals key regulons at each time point, it lacks the ability to elucidate regulon relationships and cooperativity across time.

**Table 1:** Relative reduction in MAD (RRMAD) by combinations of rRNA processing motifs under different conditions.

Motif combinations	Sporulation	Heat shock	Alternative carbon sources	DTT exposure	Nitrogen depletion
mRRPE	0.14	0.31	0.15	0.04	0.06
PAC	–	–	–	0.05	0.32
PAC-mRRSE3	–	0.34	0.25	0.15	0.25
2 mRRPE	0.24	–	–	0.41	0.11
mRRPE-PAC	0.12	–	0.56	0.63	0.40
mRRPE-PAC-mRRSE3	–	0.57	0.61	0.59	0.49
2 mRRPE-PAC	–	–	–	–	0.40
2 mRRPE-PAC-mRRSE3	0.21	–	–	–	

#### E. Comparisons of MRF to cluster analysis

**Figure 7:**
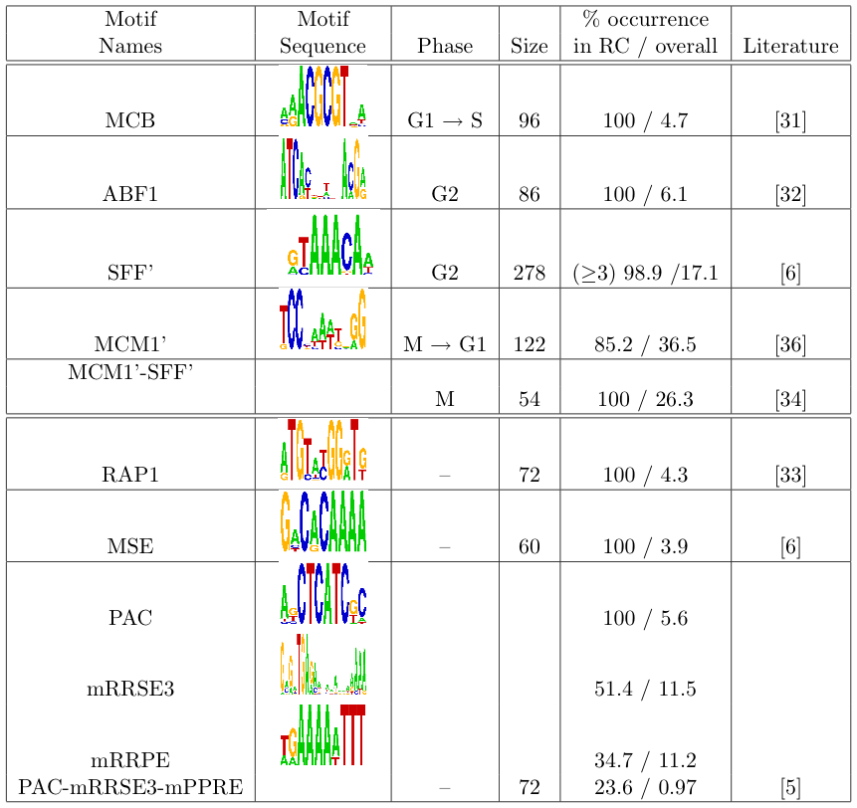
Signature motifs in identified cell cycle RCs using motifs as predictors.

We then compared our method to unsupervised clustering, which had early successes in analyzing motif-expression relationships. We applied PAM to the cell cycle expression data, and prescribed 13 clusters, matching the number of clusters used with MRF. The sizes of the resulting clusters ranged from 94 to 256, appreciably more uniform than those derived from MRF, which ranged from 54 to 599. The largest MRF cluster consists of essentially null (non-varying) genes. Cross-tabulating these two gene categorization schemes reveals that the members of this large null cluster are evenly distributed across all unsupervised clusters, potentially diluting meaningful cluster-specific information. Indeed, enrichment analysis conducted within each unsupervised cluster yields only four clusters with significant feature motifs. Moreover, the signals within each cluster are much more attenuated. The RAP1 cluster has 145 genes, but only 30.3% of them contain the actual RAP1 motif. The MCB cluster has 96 members with 28.6% MCB motif occurrence. The MCM1' cluster contains 181 genes with a 56.9% prevalence of the MCM1' motif. Lastly, the MSE cluster is comprised of 228 members, only 9.1% of which possess the MSE motifs. The stark contrast in motif enrichment strength compared to MRF (See Figure 7) is due to a lack of simultaneous evaluation of both components of regulation: motif and expression. Such limitations are inherent in unsupervised approaches and have been widely noted in the context of microarray classification / regression problems. Increasing the number of clusters does not lead to discovery of more meaningful regulatory modules (results not shown).

### Application of MRF on Cell Cycle Using Both Motif and TF Binding Data as Predictors

**Figure 8:**
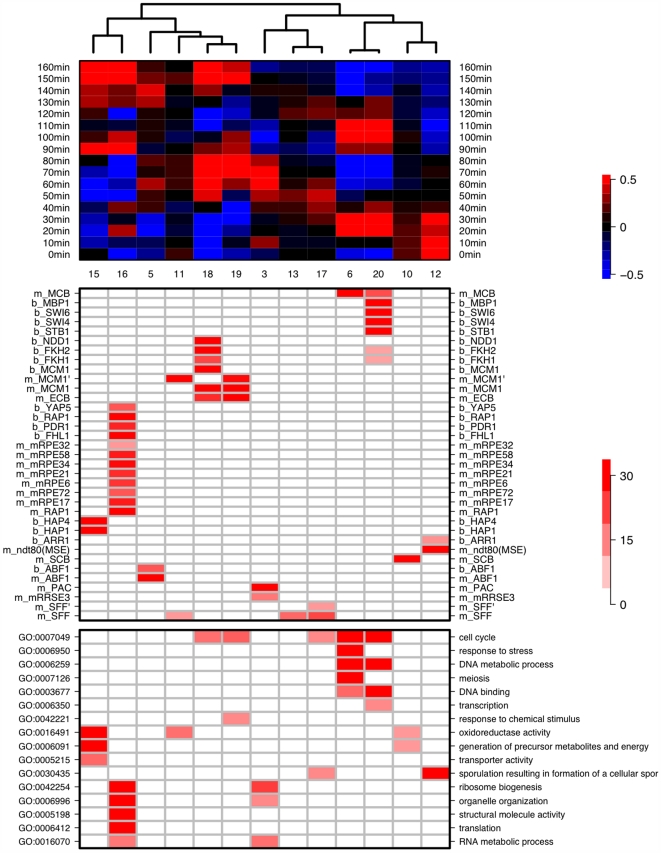
RC diagram of the cell cycle data by Cho *et al.* using both motifs and TF-binding as predictors. The top section of the graph shows the average expression profile of the genes in a specific RC, which is clustered based on Pearson correlation and average linkage. The middle section depicts signature regulons in the corresponding RC. Motif regulons have the “m_” prefix whereas TF-binding regulons have the “b_” prefix. The color red indicates enrichment 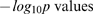 by a Chi-square test of association; the color blue corresponds to the depletion 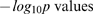. The bottom section shows enrichment of GO categories.

As a set of comprehensive binding data for 203 TFs exists [21], and as both the binding data and the cell cycle data were conducted in the same medium (YPD), it is natural to combine motif and TF-binding data as predictors and use MRF to model the cell cycle expression profile. This exercise can affirm results obtained above, which only used motif data as predictors. In addition, it might also provide insight into the relationship between motif nucleotide sequences and the actual TF binding sequences. The derived RC diagram, together with gene ontology (GO) enrichment for each RC, is displayed in Figure 8, in which motif and binding regulons are differentiated by an “m_” or “b_” prefix respectively. As expected, many motifs and their corresponding TF-binding were consistently enriched in the same RC, for instance, “b_ABF1” and “m_ABF1”, “b_MCM1” and “m_MCM1”, and “b_RAP1” and “m_RAP1” were both signature regulons in the same RCs. Again, MRF was able to reconstruct cyclic and non-cyclic time profiles, corroborating findings by using only motif-data. Below are a few noteworthy cases.

#### MCB

**Figure 9:**
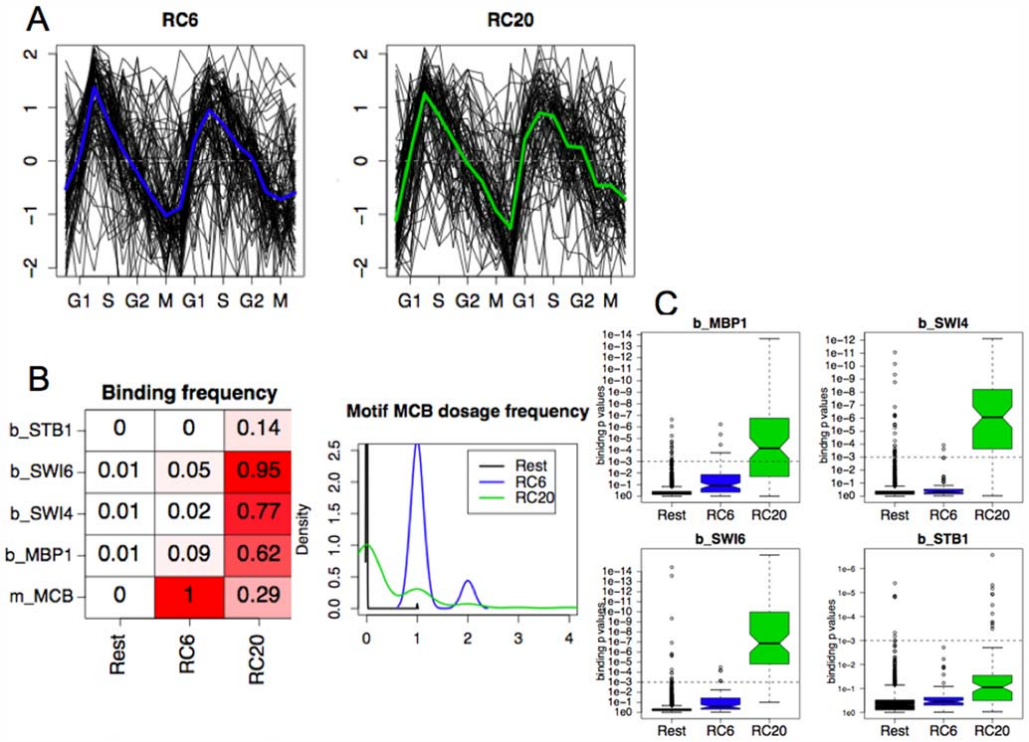
Comparisons of RC6 and RC20 uncovered in yeast cell cycle data by Cho *et al.* using both motifs and TF-binding as predictors. A Expression profiles of constituent genes. B Left: binding and motif frequency of feature regulons in the two RCs; Right: MCB motif dosages in the two RCs. C Boxplots of binding p-values of the four binding TFs comparing RC6, RC20 and the rest of the genes.

We have identified two MCB RCs (RC6 and RC20) that exhibit cyclic expression profile peaking during the G1→S transition; the expression traces of member genes from both RCs are displayed in Figure 9A. RC20 contains 79 genes, whose promoters, according to the binding data, have high occupancy of the transcription factors Swi6 (95%), Swi4 (%), Mbp1(62%) and Stb1 (14%). The Swi factor Swi6 is a cofactor for both Swi4 and Mbp1, forming SBF and MBF activators with them respectively to regulate late G1 genes. Stb1 has recently been found to also be associated with G1-specific promoters during G1-phase [39]. In contrast, RC6 has 91 genes, all of which has weak or no binding of these four transcription factors (see Figure 9B). We illustrate these differences in binding strength by boxplots of the binding p-values from RC6, RC20 and the rest of the genes in Figure 9C. Interestingly, even though RC6 genes have no demonstrated binding of the MCB binding factor (Mbp1) and its G1 transcription regulation partners (Swi6, Swi4 and Stb1), 100% of these 91 genes possess at least one copy of the MCB motif and display the cyclic transcriptional activities with the same periodicity, phase and strength as genes in RC20. Moreover, the existence of these two MCB RCs is further confirmed by the analysis of another independently generated cell cycle dataset [3]; see Text S1 Figure 7. This suggests that a subset of MCB-possessing genes are *dependent* upon the MCB motif sequence for its periodic transcription during the cell cycle, but are *independent* of known MCB binding transcription factors, indicating perhaps an alternative regulation route via one or more unknown MCB binding factors. Comparing GO category enrichment (bottom panel of Figure 8) of these two cliques shows that genes from both cliques are enriched in cell cycle, DNA metabolic process, and DNA binding, but RC6 genes are also additionally involved in response to stress and meiosis.

#### MCM1

There are two MCM1 enriched RCs, RC18 and RC19. RC18 is characterized with the association of TFs Mcm1, Fkh1, Fkh2, Ndd1 with the motif MCM1, whereas RC19 genes uniformly possess MCM1 motif, yet lack the binding of the aforementioned TFs; see Text S1 Figure 5. The phases of RC18 and RC19 genes are different, with RC19 genes peaking during mitotic exit (M→G1; [35],[36]), whereas the RC18 genes exhibit binding of Fkh1 or Fkh2 with Mcm1 and increased expression in M. These two MCM1-based RCs are also identified in the Spellman *et al.* data [3] (see Text S1 Figure 7) with the same lag in phases.

### Comparisons with Existing Methods that Model Yeast Regulon-Expression Relationships

#### A. Comparison with DREM

Except for multivariate trees, whose performance we contrasted with MRF earlier, all existing methodologies that utilize the regression framework to model yeast regulon-expression relationship and to identify pivotal regulators rely on a univariate outcome. Most of these methods operate within each time point and attempt to synthesize information across times in a heuristic manner. One recent method that is notably different and is specifically designed to model yeast time series data is due to Ernst *et al.* [11]. The authors developed a novel computational method, DREM, which uses an input–output hidden Markov model to identify regulatory networks. DREM works by identifying bifurcation points, these being places in the time series where the expression of a subset of genes diverges from the rest of the genes. Over-enrichment scores based on hypergeometric tests are then used to associate TFs with such splits. The authors provided the DREM software on their website (http://www.sb.cs.cmu.edu/drem), which we ran on the cell cycle data of Cho *et al.* [22] with default parameter settings. The output dynamic regulatory map is displayed in Text S1 Figure 6. To facilitate interpretation, we summarized the significantly enriched TFs associated with correspondingly colored nodes in colored and numbered boxes underneath the map. Consistent with the findings of MRF, DREM also identified the following regulatory circuits: (1) MCB, Swi4, Swi6 and Mbp1; (2) RAP1, Rap1 and Fhl1. It also discovered Ndd1 and Gcn4, whereas MRF assigned Ndd1 to MCM1-based RCs. Motifs or TFs that play prominent roles in the cell cycle but were not detected by DREM include MCM1 and its partners. The regulatory map output by DREM shows two major divergent paths (A and B) that can be associated with TFs. Path A (gray node/box A1) contains TFs that are known to activate periodic transcription. Path B (red node/box B1) contains Rap1 and Fhl1 based transcription at the start of the path, but then splits into two sub-paths (B3 and B4) at 130 minutes, with B3 corresponding to MCB, Mbp1, Swi6 and Swi4 (dark gray box). Note that the same TFs are also similarly enriched in A2. Comparing paths A and B, it can not be readily reconciled why two sub-paths (A2 and B4), divergent from the outset, are associated with the same set of enrichment TFs. In addition, none of the expression traces of the different paths exhibit cyclic behavior, and none of the identified motifs or TFs can be confidently assigned to a phase in the cell cycle. This suggests modeling via bifurcations is perhaps not the most suitable approach for investigating the cell cycle or periodic series in general.

#### B. Comparison with Zhang *et al*

Another notable improvement over single time point modeling is proposed by Zhang *et al.* [13]. Their method defines the regression loss function as weighted sum of losses over the principal component (PC) scores or linear contrasts of the initial outcome variables. The use of PCs instead of single time point samples effectively captures meaningful time-dependent structure in the data. We refer to the projection-based regression method of Zhang *et al.* [13] as PBR hereafter. Zhang *et al.* [13] applied PBR to the cell cycle data of Spellman *et al.* [3]. To properly compare MRF with PBR, and to investigate whether MRF can obtain reproducible results on two independent data sets interrogating the same biological conditions, we ran MRF on the cell cycle data of Spellman *et al.* [3]. The derived RC diagram is displayed in Text S1 Figure 7. The most prominent cyclic motifs, including MCB, SWI5, MCM1, and SFF, are identified by both methods. MRF additionally identified ABF1, consistent with the findings in Cho *et al.*, but missed by PBR. However, the most noteworthy differences between the results of the two methods concern non-cyclic motifs and motif interactions. MRF is able to identify regulatory circuits of RAP1 and multiple mRPEs, which regulate ribosomal protein genes, and those of mRRPE, PAC and mRRSE3, which are involved in ribosomal RNA processing. The time-dependent expression trend of these genes increases with time, after initial dampening, and so should strongly correlate with the third PC projection used by PBR. The failure to identify patterns that do not correlate with the strongest PCs showcases problems with PCA regression whereby key modes of variation may not correspond to (leading) PC directions.

#### C. Comparison of MRF findings on cell cycle data from Cho *et al.* and Spellman *et al*

RC diagrams from the two cell cycle data sets, (Cho *et al.* [22] and Spellman *et al.* [3]), are displayed in Figure 8 and Text S1 Figure 7. The results are remarkably consistent in terms of the RCs elicited by MRF. Most interestingly, two MCB related RCs emerged from the Spellman *et al.* data, one characterized by the MCB motifs and the other characterized by Mbp1 binding, similar to their counterpart RCs uncovered using the Cho *et al.* data. These two RCs share very high percentages of common genes – 94% and 82% respectively – between the two datasets. This reproducibility reinforces our hypothesized existence of an alternative, and currently unknown, MCB-binding pathway. To further examine whether constituent genes in the RCs characterized by the same regulons between these two data sets resemble one other, we tabulated percentage of common genes among select RCs in Text S1 Figure 8. The tabulation confirms that the similarity between RCs featuring the same regulons of the two data sets is >70% whereas it is <10% between RCs featuring different regulons.

### Application of MRF to Sporulation and Stress Conditions

Unlike the yeast cell cycle data, where the expression data were performed under the same biological condition as the binding data, stress and sporulation conditions have only either no, or very sparse (≤10 TFs), TF binding information. We therefore used only motifs as predictors.

#### A. RCs in sporulation

**Figure 10:**
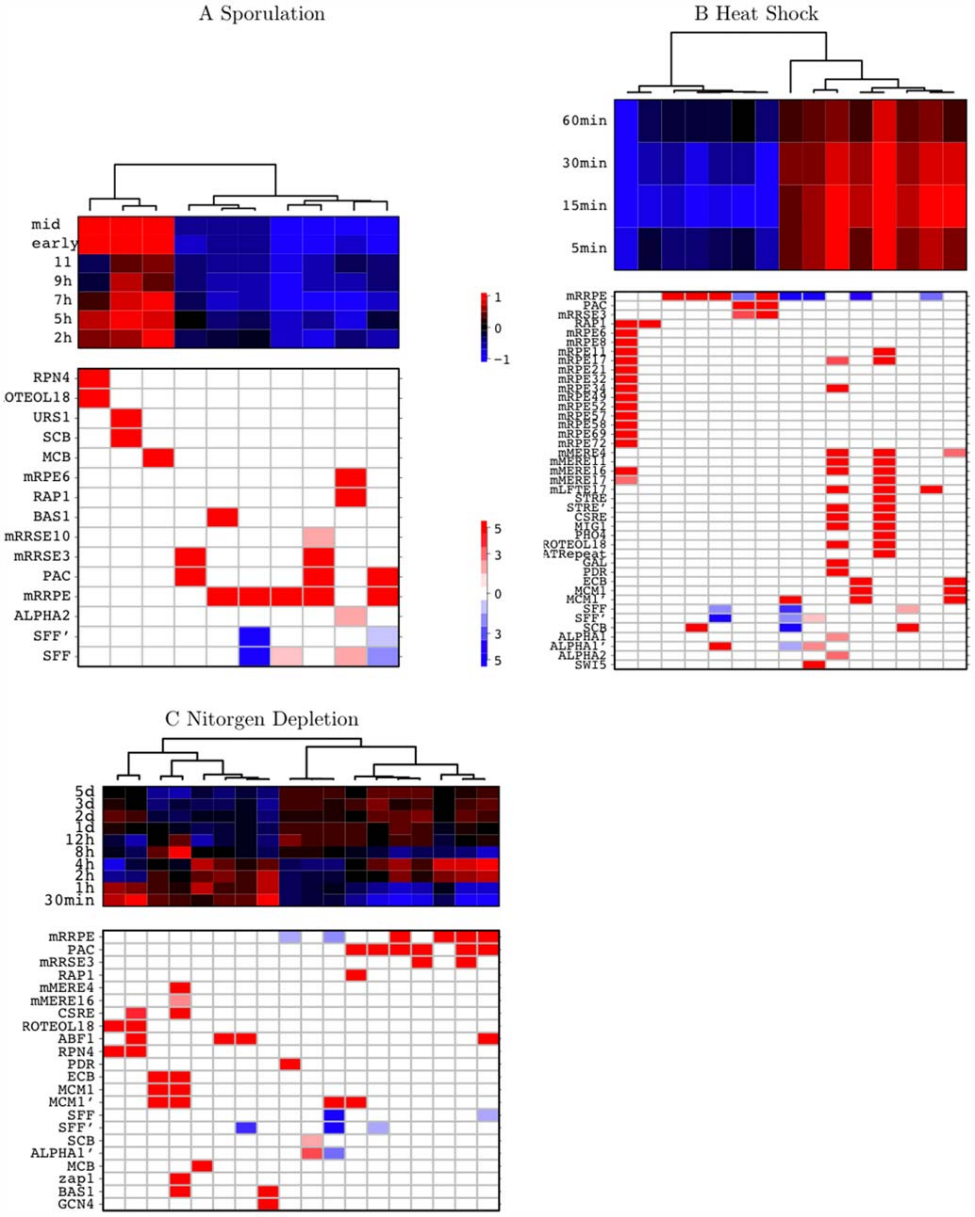
RC diagrams for (A) sporulation (B) heat shock and (C) nitrogen deplection. The top section shows that dendrogram of hierarchical clustering of the average expression profiles (in log_2_-ratios) within each RC based on Pearson correlation and average linkage. The bottom section depicts signature motifs in the corresponding RC. The color red indicates enrichment 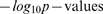 by a Chi-square test of association; the color blue corresponds to the depletion 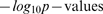. The color bar at the lower right hand side is in 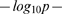 scale and the color signals the direction of the test.

The RC diagram of the sporulation data set [23] is clustered into two distinct groups that exhibit increased and decreased expression upon entering sporulation respectively (Figure 10A). The direction of the transcription response to sporulation is clearly associated with the presence of the mRRPE motif, which is the rRNA processing element. The expression of genes that possess the mRRPE motifs, or combinations of the rRNA synthesis/processing motifs (PAC, mRRPE, mRRSE3 and mRRSE10), is repressed throughout the sporulation process. Such repression is also seen in genes that have the RAP1 motif. This corroborating evidence of a decline in gene expression relating to the production of the ribosomal machinery may be the result of a growth respite caused by nitrogen starvation in order to trigger the sporulation process. Interestingly, we have identified RCs of different combinations of this group of rRNA-related motifs: mRRPE, PAC-mRRPE, PAC-mRRSE3 and mRRPE-PAC-mRRSE3. The composition of these combinations have differing consequences for the profiles, and magnitudes, of expression changes further highlighting the combinatorial transcription control of rRNA processing. Among the genes that are induced upon entering sporulation three distinctive RCs emerge: URS1-SCB, MCB, and RPN4-mPROTEOL18. This is consistent with previous studies that suggest the involvement of cell cycle (MCB and SCB; [6],[33]) and stress (RPN4 and mPROTEOL18; [6]) motifs in sporulation. URS1 is the binding site of the Ume6/lme1 complex which is the major transcriptional regulator of genes involved in early phase meiosis [23].

#### B. RCs in stress conditions

To further reveal conditional-specific RCs, we also investigated four different stress conditions: heat shock, nitrogen depletion, DTT exposure, and steady-state growth on alternative carbon sources [24]. The resulting RC diagrams are displayed in Figure 10B and 10C and Text S1 Figure 9. Similar to sporulation, all four stress conditions invoke diametrical responses between ribosome-related RCs and stress-specific RCs, evidenced by the separation of these two groups into two opposing branches of the hierarchical tree built based on expression profiles. The ribosome-related clusters again include two groups of co-operative genes involved in ribosome production: (i) RAP1 and multiple mRPEs (MIPS ribosomal protein elements), that regulate the transcription of ribosomal protein genes; and (ii) mRRPE, PAC, mRRSE3 and mRRSE10, that are involved in ribosomal RNA processing. However, the response to reduce ribosome production elicited by these stress stimuli differs in the speed: heat shock triggers a much more rapid reaction than both nitrogen depletion and DTT exposure. Also notable is the emergence of two exclusive RAP1 RCs in heat shock and DTT exposure, one of which involves only RAP1, whereas the other features many different mRPEs in addition to RAP1. Interestingly, in sporulation, we also identified a RAP1 RC that included both RAP1 and mRPE6 motifs. Consistent with findings in Pilpel *et al.* [33], mRPE6 appears to be a potential interacting partner of RAP1 in the process of regulating ribosomal protein production across different conditions. The RCs characterized by the different stress-specifc motifs have induced expression to combat unfavorable exterior stimuli. And again, the induction triggered by heat shock is much more prompt than that for the other stress conditions. Among those motifs that contribute to increased expression many functional categories are involved, underscoring the massive changes in metabolism and development that the cells enlist to withstand adverse conditions. These include (i) stress-related motifs: RPN4, mPROTEOL18, STRE and CSRE; (ii) energy-related motifs: mMEREs and mLFTE17; (iii) cell cycle-related motifs: MCM1', MCB and SCB; and (iv) amino-acid biosynthesis related motifs: BAS1 and GCN4.

#### C. Combinatorial control of the rRNA processing motifs

**Figure 11:**
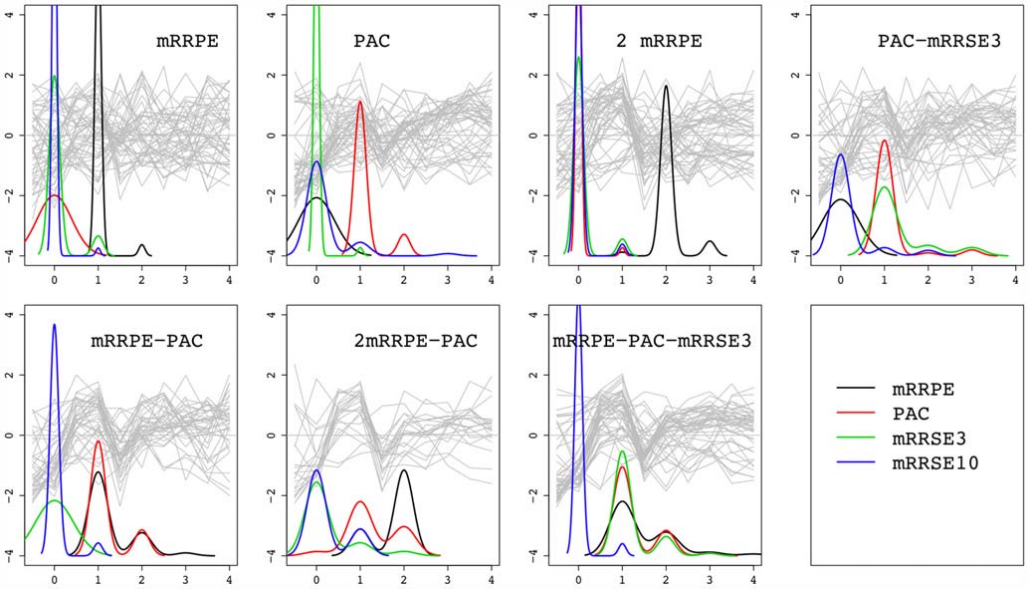
Combinatorial controls of rRNA processing motifs in nitrogen depletion. Each panel corresponds to an RC that involves one or more motifs from the rRNA processing motif group that include mRRPE, PAC, mRRSE3 and mRRSE10. Gray traces are expression profiles of the gene targets allocated in a specific RC. The black, red, green and blue lines are densities of motif counts for mRRPE, PAC, mRRSE3 and mRRSE10 respectively. The motif counts can be read off from the X axis.

The four rRNA processing motifs, mRRPE, PAC, mRRSE3, and mRRSE10, have exhibited the capacity to impart fine control on transcription in a combinatorial fashion. We investigate next how different combinations of these four motifs affect gene expression and whether this influence is condition-specific. To this end, we examined conditions that have at least 2 different combinations of the rRNA processing motifs. These include all conditions except for the cell cycle. For each data set, and each RC, we calculated its percent relative reduction in dispersion compared to the overall dispersion of the entire data set, with dispersion measured using median absolute deviation (MAD). This summary, termed RRMAD, is displayed in Table 1. By definition, a synergistic event among constituent motifs will lead to more cohesive expression profiles and a greater reduction in expression dispersion, and therefore a higher RRMAD. To visualize this effect we display gene expression traces within each combination group in nitrogen depletion, with densities of doses of the four motifs superimposed in Figure 11. Table 1 exemplifies the complexity of combinatorial control as arising from the following effects.

1. *Condition-dependent mRRPE-PAC synergistic effect*. Interestingly, among the conditions surveyed, only in sporulation is a concerted effect between PAC and mRRPE not observed. Specifically from Table 1, the RRMAD for the mRRPE RC in sporulation is 0.14 whereas it drops slightly to 0.12 in the RC that contains both PAC and mRRPE. This is in stark contrast to all other conditions. For instance, in DTT exposure, RCs that contains a single motif, either mRRPE or PAC are very dispersed with RRMAD smaller than 0.1, however for genes that possess both PAC and mRRPE, their expression profiles have sharply increased coherence with RRMAD larger than 0.6, suggesting high cooperativity between these two motifs.

2. *Condition-dependent mRRPE dosage effect*. In sporulation, DTT exposure and nitrogen depletion, we have observed dosage effects of mRRPE. It is most striking in DTT exposure, evidenced by the large increase of RRMAD from 0.04 for a single copy to 0.41 for two copies of mRRPE. In nitrogen depletion the dosage effect of mRRPE, while less apparent, appears to be time dependent with later time points exhibiting more uniformity with an additional copy of mRRPE, but earlier time points being less affected (see Figure 11).

3. *Condition-dependent mRRPE, PAC and mRRSE3 main effect*. Heat shock is the only condition, where genes containing only one copy of mRRPE have relatively tight expression, suggesting an important role of mRRPE in heat shock. Compared to mRRPE, effects of PAC and mRRSE3 are attenuated, with the exception of PAC in nitrogen depletion. In fact, a single copy of mRRPE has no direct influence on gene expression in nitrogen depletion, yet possessing a copy of PAC reduces RRMAD by more than 30%, suggesting that PAC by itself is sufficient to exert expression response in the event of nitrogen depletion. But overall, the results here show that these three motifs appear to exert most of their influence through co-operative activity, rather than via sole (main) effects.

4. *mRRSE3 and mRRSE10*. The mRRSE3 motif is in the same RC with PAC under the heat shock and DTT exposure conditions, and with PAC and mRRPE in all conditions examined, but does not appear to have any synergistic effect with mRRPE only. The motif mRRSE10 is enriched in RCs in sporulation, DTT exposure, and alternative carbon sources. However, unlike the other three motifs, none of these RCs has a 100% occurrence of at least one copy of mRRSE10.

In summary, the combinatorial control of rRNA by the four rRNA processing motifs presents an elegant testimony to the complexity of transcriptional regulation governed at various levels by different motifs, different motif combinations, different motif dosages, and under different biological conditions. A recent paper by Boorsma *et al.* [1] has also suggested the importance of PAC and mRRPE in yeast transcription regulation and posited that they may serve as NC2-dependent core promoter elements.

#### D. Gene targets of the RAP1 and mRPEs RCs

Pairwise comparisons of the genes in the RAP1 related RCs in the alternative carbon sources, DTT exposure, and heat shock stress conditions show that a majority of them are common targets. In fact, between 53% and 71% of the genes are shared between any two conditions. There are 41 genes with known function (3 have unknown function) that are present in all 3 conditions, all but one of which encode ribosomal protein subunits, with the exception being SST2, whose N terminus regulates stress response [40]. By the nature of RC derivation the ribosomal proteins in RAP1-mRPE cliques have, on average, six copies of mRPEs. This is much higher that the ribosomal proteins in the RAP1 clique, which typically possess only one mRPE copy. However, the higher dosage of mRPEs does not lead to much tighter expression control, as evidenced by the gene expression traces of these two different cliques for heat shock, shown in Text S1 Figure 10. This suggests that the RAP1 motif is dominant in the repression of ribosomal proteins in response to environmental perturbations, and that the mRPE motifs have only accessory roles.

#### E. Gene targets of the MCB RCs

We identified the MCB regulatory clique in two conditions in addition to the cell cycle: sporulation and nitrogen depletion. These three MCB RCs all have tight, co-ordinated expression profiles, and the relative reduction in MAD is 33% (nitrogen depletion), 35% (cell cycle) and 39% (sporulation). Pilpel *et al.* [33] and Bussemaker *et al.* [6] also noted the correlation between the MCB motif and expression in meiosis. We have observed that 38% of the gene targets of the MCB RC in sporulation are involved in DNA metabolic processes, and this includes various CDCs (CDC6, 7, 9, 21, 45), DNA polymerase subunits (POL1, 2, 12, 30, 31 and 32), various RADs (RAD17, 27, 53 and 54), and subunits of replication factors A and C (RFA1, RFA2 and RFC4). This is perhaps not surprising given the prominent role of MCB in regulating DNA synthesis in the cell cycle. While MCB is an established regulator in mitotic gene expression, the mechanism of its involvement in sporulation / meiosis is not completely understood. Futcher [41] speculated that as the known positive regulator of MBF is Cln3, which is antagonistic to meiosis, these DNA synthesis genes, marked by MCB sites, could be regulated very differently in meiosis. Raithatha and Stuart [42] similarly suggested that an alternative MCB binding factor may exist. Since we have uncovered two MCB-based RCs (RC6 and RC20) in the cell cycle, and RC6 has no TF binding signals whereas RC20 features strong binding from Mbp1 and its partners, we compare the constituent genes in the MCB RCs in sporulation and nitrogen depletion with those in RC6 and RC20 in the cell cycle. We have observed that the MCB RC in sporulation shares 76% common genes with RC6 and only 26% common genes with RC20; similarly the MCB RC in nitrogen depletion shares 97% common genes with RC6 and 40% common genes with RC20. This is consistent with our GO enrichment analysis that shows RC6 genes are additionally enriched in response to stress and meiosis compared to RC20 (see bottom panel of Figure 8). All this evidence strongly suggests that an alternative MCB-binding pathway, that does not involve Mbp1, is active in the cell cycle, sporulation and nitrogen depletion.

## Discussion

In this paper, we propose a novel, random forest based algorithm to identify condition-specific, regulatory cliques that feature genes that are co-expressed and co-regulated in the yeast *S. cerevisiae*. As we have shown, our method, multivariate random forests (MRFs), enjoys the following advantages over many existing methods in modeling transcriptional regulation: (i) it simultaneously models both regulon-expression relationships and combinatorial regulation; (ii) it identifies high-order regulatory networks without being compromised by combinatorial explosion; (iii) it handles both univariate and multivariate responses; (iv) it readily identifies motifs affecting the whole spectrum of experimental conditions and those involved in only a subset of conditions.

MRF builds upon widely used regression tree techniques. This popularity arguably derives from trees' interpretability (enhanced by visualization of tree schematics), avoidance of parametric assumptions, and flexibility in accommodating large numbers of covariates. Multivariate response regression trees were first proposed by Segal [17], and Phuong *et al.* [10] used this methodology to study yeast expression regulation. As we have detailed, the advantage of applying tree techniques in the context of regulation problems is that genes are partitioned into homogeneous groups with respect to both motifs and expression. However, well known limitations of single trees diminish their utility in elucidating regulatory networks as defined via motif interactions. Of particular concern is that the greedy algorithm employed in tree construction precludes assessment of an extensive repertoire of motif interactions. Indeed, Phuong *et al.* [10] recovered only *one* pair of interacting motifs, mRRPE-PAC, in sporulation, highlighting this potential deficiency of using single trees.

Our MRF algorithm rectifies this problem for multivariate trees in precisely the same manner as the original random forest approach [19] improved univariate trees. The injection of randomness and the formation of an ensemble of multivariate trees produces a greatly increased inventory of candidate motif interactions, including those lacking strong main effects. To fully realize this potential we have devised a bottom-up approach that utilizes the output proximity matrix to derive cohesive regulatory cliques. We have shown that our algorithm for deriving regulatory cliques is both stable and sensitive. The latter is evidenced by the novel revelation of dosage effects of the SFF' and mRRPE motifs, and the refined dissection of combinatorial control of the rRNA processing motifs under different conditions. As illustrated in the RC diagrams in Figures 4 and 10, MRF is effective in identifying synergistic motifs, notably recovering those without strong main effects. Examples here include the partnerships of RPN4-mPROTEOL18 (in sporulation and nitrogen depletion), PAC-mRRSE3 (in heat shock, alternative carbon sources, and DTT exposure), mMERE4 and mMERE16 (in DTT exposure, heat shock, and nitrogen depletion), and BAS1-GCN4 (in nitrogen depletion). The RCs extracted display a dominance of rRNA and ribosomal protein motifs. This is as anticipated, in line with special physiological characteristics that define yeast cells. It is estimated that in a rapidly growing yeast cell, 60% of total transcription is devoted to ribosomal RNA, and 50% of RNA polymerase II transcription and 90% of mRNA splicing are devoted to ribosomal proteins [43].

For the cell cycle data, we have used both motif-based predictors and motif-and-TF-binding-based predictors. Even though TF-binding reflects the actual TF binding level, and likely has fewer false positives than motif counts, we found that there are merits in including motif sequence information. In particular, it facilitates hypothesis generation as many motifs have no known TF binding information. For instance, as in Boorsma *et al.* [1], we have uncovered the importance of PAC and mRRPE motifs in yeast transcription regulation even though currently there are no known TF bindings to either motif. Another example is MCB: even though Mbp1 is known to occupy the MCB motif, we have shown here that there could be another unknown TF that regulates genes through binding to the MCB motif, this proposition also having been raised by Raithatha and Stuart [42].

To model the relationship between regulatory elements and gene expression, our method requires a set of known motifs or known TF binding information. It is however potentially useful in other settings as well. For instance, expression quantitative trait loci (eQTL) studies that seek to elucidate associations between gene expression and marker genotypes at specific genetic loci can naturally be modeled via MRF. Moreover, gene regulation through microRNA bindings can be investigated by linking microRNA and gene expression data using MRF. These, however, represent more complex and intricate relationship than yeast gene regulation, and the utility of MRF in these settings is the subject of future research.

## Materials and Methods

### Data Preprocessing

#### Microarray data

We used the *S. cerevisiae* microarray data sets in the cell cycle [22], sporulation [23] and stress conditions in heat shock, nitrogen depletion, DTT exposure, and steady-state growth on alternative carbon sources [24]. Except for the cell cycle data that used one-channel Affymetrix arrays, all other experiments employed two-color cDNA arrays. The first four were all time course studies with 17 (cell cycle), 8 (sporulation), 4 (heat shock), 10 (nitrogen depletion), and 7 (DTT) expression measures per gene. There were 6 differing carbon sources. Following Tavazoie *et al.* [4] and Phuong *et al.* [10], we selected the top 3000 most-variable genes from each data set and applied gene-wise normalization. This was carried out by subtracting the mean and dividing by the standard deviation across all samples from the expression level of each gene.

#### Motif data

We used the established DNA motif database from Pilpel *et al.* [33] as our motif covariates. This set of motifs contain 37 known motifs and 319 unknown motifs, derived by applying AlignACE [44] to the upstream regions of genes in MIPS [45] functional categories. For each motif, we used ScanACE [44] to count the number of times it appears in the promoter regions of the genes.

#### Binding data

We used the TF binding data of Harbison *et al.* [21]. The data contain ChIP-chip results for 203 TFs performed in rich medium (YPD). The binding information were dichotomized using the binding p-value threshold 0.001. A TF was considered to be binding to a gene if the binding p-value reported by the authors was smaller than 0.001.

### Multivariate Regression Tree

Suppose 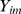 (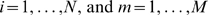) and 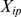 (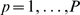) are response and predictor variables respectively. Here 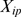 are counts of the 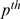 motif for the (upstream region of the) 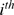 gene, and 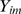 and expression levels for the 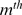 condition (time point) for the 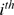 gene. We use the regression tree paradigm to estimate the functional relationship between the predictor variables (motif counts) and the response variables (gene expression).

The regression tree framework is described in Breiman *et al.* [15]. Regression tree construction involves four components: (1) A set of binary (yes/no) questions, or splits, phrased in terms of the covariates that serve to partition the covariate space. The sub-samples created by assigning cases according to these splits are termed nodes. A node that does not have any descendant nodes is a terminal node. (2) A node impurity measure, typically relating to variance in the regression context. (3) A split function, 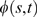, that can be evaluated for each split 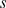, of each node 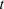. The best split, which optimizes 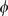, is such that the response distributions in the resultant children nodes are most homogenous amongst all competing splits, with homogeneity assessed via the impurity measure. (4) A means for determining appropriate tree size.

Consider a node 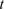 containing a sub-sample of cases 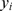, with corresponding covariates 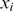. We aim to partition 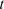 into two child nodes, a “left” node 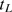, and a “right” node 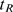. Our motif counts 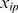 are ordered covariates so that (default) allowable splits are order-preserving binary cuts of the form 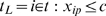, 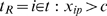 as the (motif count) cut-point 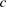 ranges over all possible values resulting in distinct 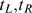. Following Segal [17], we define the node impurity measure as
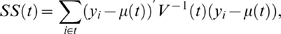
where 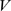 is the covariance matrix of 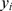, and 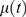 is the mean of 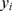 in node 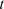. Here, to simplify computation, we impose 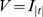 (the identity matrix) for all 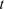. For each motif, we evaluate all possible splits according to the split function,
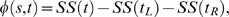
with the split 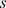 that maximizes 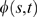 being selected to partition node 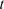.

The prediction for each leaf of a constituent regression tree here is the vector of mean expression values at each time point/condition for genes reaching that leaf.

### Multivariate Random Forest

Breiman [19] has demonstrated that consequential gains in prediction accuracy can be achieved by using (large) ensembles of trees. Each tree is constructed from a bootstrap sample drawn with replacement from the full data set. A valuable by-product of this approach is that those cases not sampled, termed out-of-bag (OOB), provide a ready made test set, enabling unbiased estimation of prediction performance without recourse to cross-validation. Additionally, instead of determining the optimal split of a given node of a (constituent) tree by evaluating all allowable splits on all covariates, as is done with single tree methods, a random subset of the covariates is used. The size of this subset, mtry, constitutes the primary tuning parameter of the random forest procedure. Beriman [19] argues that random forests enjoy exceptional prediction accuracy for a wide range of settings of mtry. Here, we used ensembles of size 1000, minimum terminal node size 20, and the recommended value of mtry, which is the square root of the number of covariates. Results were largely insensitive to varying these quantities. We implemented our MRF algorithm based on the C code from the R package *r*andomForest. R functions and scripts are available on request.

#### Proximity matrix

The interpretability of a random forest is not as straightforward as that of a single regression tree, since we are unable to readily visualize the ensemble. Thus, additional interpretative tools have been advanced for random forests. One such, proximity measures, are valuable since they capture how cases/genes relate to each other, and so are revealing about nature of influential splits. For each tree in the forest ensemble all the data (training and OOB) are run down to their assigned terminal node as dictated by the split sequence. If genes 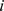 and 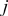 are both assigned to the same terminal node, then the proximity value, 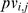, between 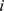 and 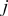 is incremented by one. This process is repeated for each tree in the forest, with proximity values normalized by dividing by the number of trees. The proximity matrix is then 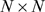 matrix of 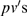, where 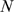 is the number of genes.

#### Variable importance measure and its significance level

For each tree, the mean square error (MSE) on the OOB data is computed. Then the same computation is performed after permuting each variable. The difference between the two MSEs, averaged over all trees and normalized by the standard error, provides a variable importance summary. To assess the significance of variable importance, we permute the rows of the response (gene expression) matrix a pre-specified (e.g. 100) number of times. The permutation generates data under the null hypothesis of no association between the regulons and gene expression. For each permuted data set, we build a multivariate random forest and calculate the variable importance measures for each motif. This gives rise to a distribution of variable importance for each regulon and enables the computation of its permutation *p-* value. This collection of *p-* values are then adjusted using the false discovery rate (FDR) control procedure proposed by Benjamini and Hochberg [30].

### Identifying Regulatory Cliques (RCs)

#### Derivation of RCs by *guided* clustering using PAM

Our *guided* clustering analysis utilized PAM (partition around medoids; [20]) to identify small and tight RCs. Each RC should contain genes that are co-expressed under at least a subset of the experimental samples, as determined by the expression data, and share the same transcription regulator binding evidence, as determined by the motif/TF binding data. The proximity matrix provides a natural similarity measure to input into a clustering algorithm in order to obtain these RCs. We chose to use the PAM clustering algorithm because of its robustness properties [20]. The algorithm first computes *k* representative objects, called medoids. The goal is to minimize the sum of distances (1 - similarity) of all observations to their closest medoid. Accordingly, the objective function is specified as 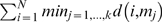, where 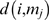 denotes the distance between observation (gene) 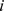 and medoid 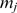. The algorithm first selects an initial set of medoids. Then the objective function is minimized iteratively by replacing one medoid with another until convergence. PAM requires the number of clusters parameter, 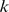, to be prescribed *a priori*. Several methods have been advanced for estimating this parameter [20],[46],[47]. However, such estimation is especially challenging in array data settings. In part this reflects the fact that the underlying genetic interactions in eukaryotic organisms are so complex that defining a precise number of exclusive and exhaustive gene clusters is misplaced [48]. Our goal in guiding clustering process is to recover (a maximal number of) coherent, tight clusters of genes resulting from specific motif-expression relationships, arising against a noisy background. To this end, we impose that the size of the smallest resultant cluster should exceed the terminal node size as specified by the multivariate random forest algorithm. Accordingly. we run PAM with a series of increasing 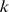 values and select the largest, 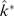, that does not violate this constraint. For each of these 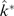 clusters we use the Kolmogorov - Smirnov (KS) test to examine whether there exists at least one phase / sample in the experiment where the expression levels of its member genes are significantly different than all other phases / samples. Clusters not displaying such differences typically have flat expression profiles; i.e. are not variable across experimental conditions. These are labeled as “null” clusters, and are excluded from further analysis. The remaining clusters constitute our RCs.

#### Identification of signature regulons of each RC

To describe each RC cluster, and reveal defining splits (motif interactions) that lead to the distinctive RC expression pattern, we seek to identify corresponding signature regulons. For each RC, we test its association with the presence of each candidate regulon. Although we used ordered motif counts in tree construction here, for simplificity, we dichotomize the counts as present (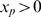) and absent (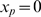). This together with the dichotomization of genes into residing in the RC or not results in a 2×2 contingency table for each RC and each regulon, on which we perform two one-sided Chi-squared tests to test for enrichment and depletion of the regulon. We employ stringent criteria in defining signature motifs, declaring significance only if the motif occurs is more than 20% of the genes and the Bonferroni adjusted Chi-square *p* value is less than 0.05.

#### Calculation of RRMAD

RRMAD is a measure that we use to quantify the amount of dispersion reduction in an RC compared to the overall null dispersion. Dispersion is measured using multivariate median absolute deviation (MAD) for robustness considerations. To compute RRMAD for the 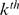 RC with constituent gene expression 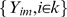, we first derive its median expression profile 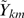, and then compute the MADs from the median profile, 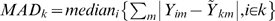. We calculate 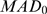 by treating the entire data set as a single. RRMAD for the 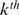 RC is then derived as 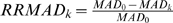.

## Supporting Information

Text S1 Supplementary Information
(1.99 MB PDF)
