# Supplementary material for: Identification of Yeast Transcriptional Regulation Networks Using Multivariate Random Forests
Source: PLoS Comput Biol. 2009 Jun 19;5(6):e1000414. doi: 10.1371/journal.pcbi.1000414 (PMC2691601; doi:10.1371/journal.pcbi.1000414)
Supplement: Text S1 — Supplementary Information (1.99 MB PDF) [file pcbi.1000414.s001.pdf]

Supporting Data: Identifying regulatory networks using multivariate random forest

Yuanyuan Xiao Mark R. Segal

January 28, 2009

Department of Epidemiology and Biostatistics,
Center for Bioinformatics and Molecular Biostatistics,
University of California, 185 Berry Street, Lobby 4, Suite 5700,
San Francisco, CA 94107, USA.

(a) Multivariate tree sample 1 (b) Multivariate tree sample 2 (c) Multivariate tree sample 3

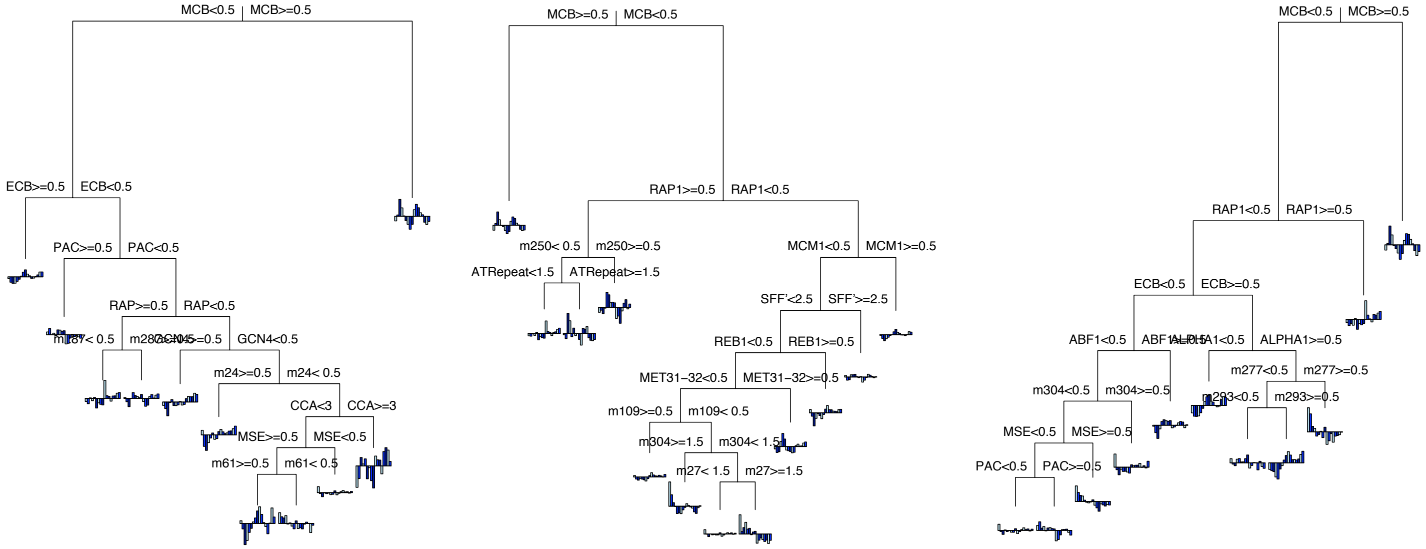

Figure 1: (a-c) Multivariate trees built using 2/3 of samples to highlight the instability of a single tree.

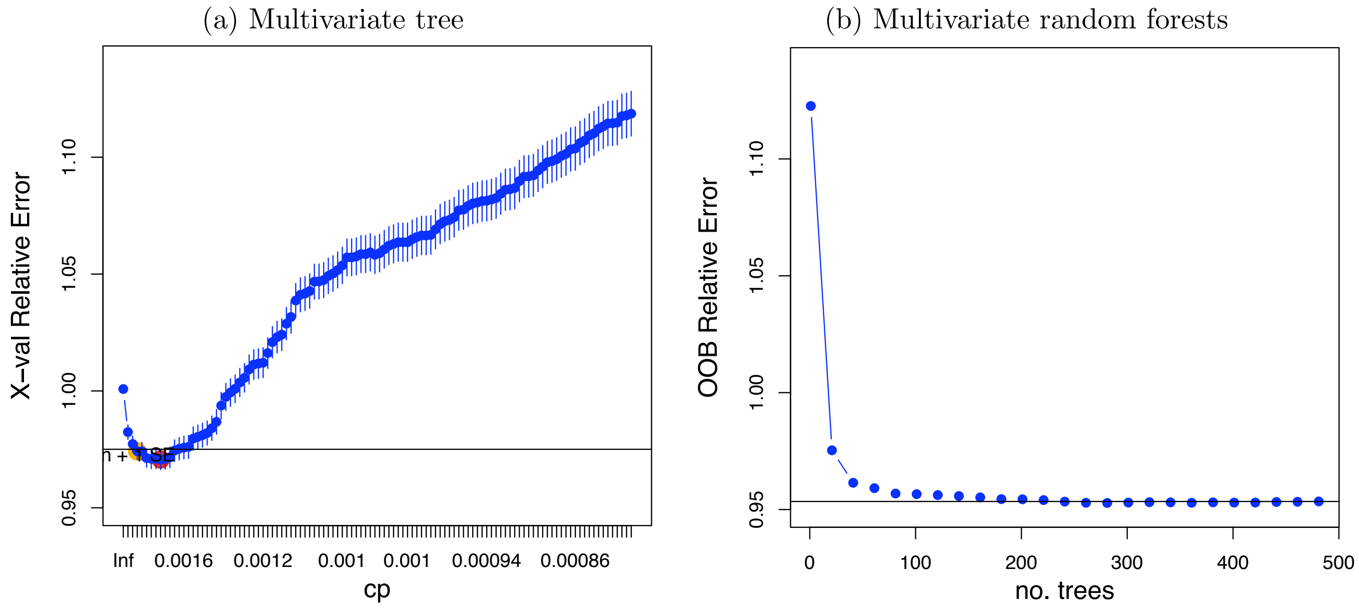

Figure 2: Prediction performance comparison between a single multivariate tree and multivariate random forests.

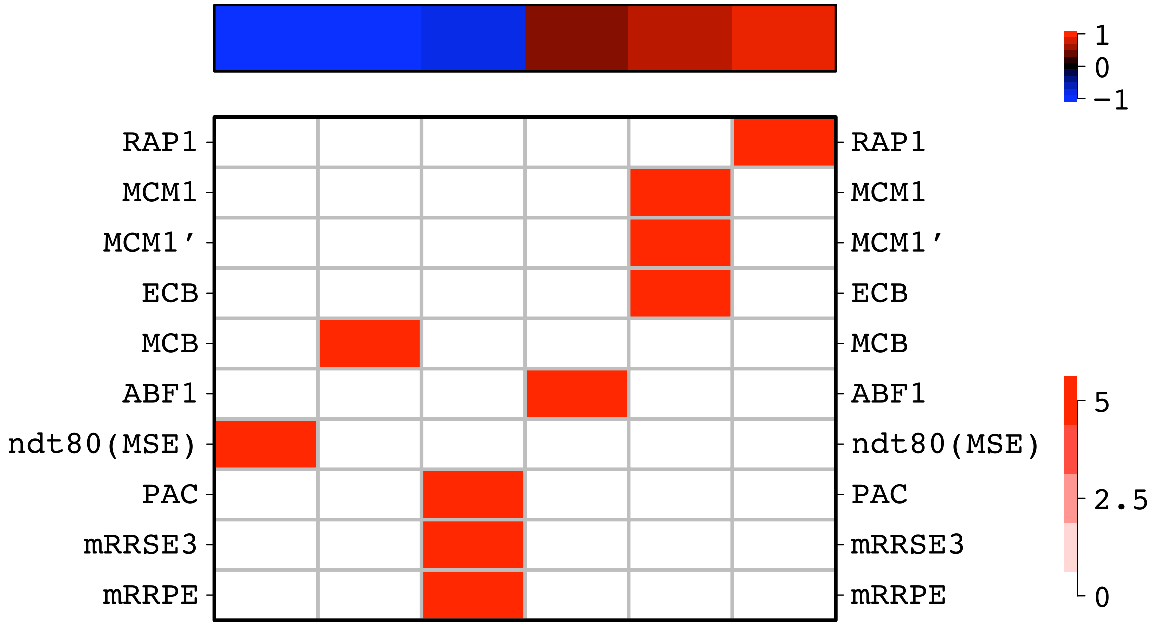

Figure 3: RCs of cell cycle derived using the 1st PC from PCA analysis of the expression matrix as the response variable.The top section of the graph shows the average scores of the genes in the RC. The bottom section depicts signature motifs in the corresponding RC. The color red indicates enrichment $-log_{10}p-$values by a Chi-square test of association; the color blue corresponds to the depletion $-log_{10}p-$values. The color bar at the lower right hand side is in $-log_{10}p-$ scale and the color signals the direction of the tes

Table 1: Top 10 importance measures for each time point identified by URF.

0min	10min	20min	30min	40min	50min	60min	70min	80min
SFF'	SFF'	MCB	MCB	SFF	SFF	SFF'	MCB	MCB
SFF	SFF	SFF'	MCM1'	SFF'	SFF'	MCB	MCM1	SFF'
MCB	ALPHA1'	SFF	MCM1	MCM1'	RAP1	SFF	MCM1'	MCM1
m304	ABF1	SCB	MSE	ECB	SWI5	PAC	ECB	RAP1
ECB	m285	ABF1	m90	m27	m310	MCM1'	SFF	SFF
MCM1'	ALPHA1	m320	ECB	m304	MET31-32	m304	ALPHA1'	ECB
m190	PAC	MCM1'	SFF'	MET31-32	m304	mRRPE	SFF'	MCM1'
MCM1	MSE	m90	SFF	MCM1	ABF1	ALPHA1'	m304	SWI5
ABF1	m310	ALPHA1'	MIG1	MIG1	MCM1'	ABF1	ALPHA1	SCB
m310	Ume6	m252	RPN4	m293	m254	MCM1	SCB	CCA
	90min	100min	110min	120min	130min	140min	150min	160min
	RAP1	MCB	MCB	SFF'	SFF'	MCB	MCB	MCB
	SFF	PAC	MSE	mRRPE	SFF	SFF'	SFF'	m304
	SWI5	SWI5	MCM1'	SFF	MCM1'	m293	SFF	SFF'
	SFF'	SFF	SFF'	MCM1	m260	SFF	ECB	SFF
	m304	ALPHA1'	m218	ALPHA1'	ALPHA1'	m310	MCM1'	MCM1'
	MCB	SFF'	m193	RAP1	m314	ALPHA1'	GCN4	SCB
	ALPHA1'	ALPHA1	m293	m289	m287	m57	m310	m271
	MCM1	MSE	ALPHA1'	ALPHA2	m289	m285	m133	PAC
	ECB	Ume6	SFF	ECB	MCM1	m_LFTE17	m293	RAP1
	STRE'	m293	ECB	m293	m105	CCA	SCB	m310

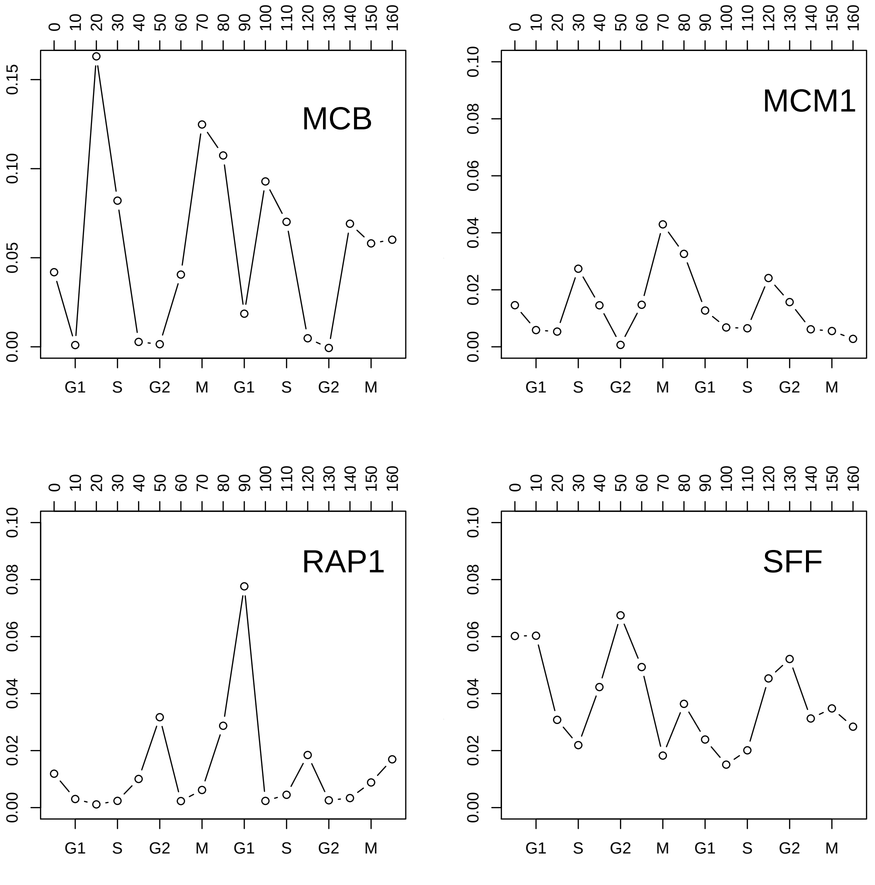

Figure 4: Normalized variable importance measures for MCB, MCM1, RAP1 and SFF' derived by applying univariate random forests for each time point of the yeast cell cycle data by Cho *et al.*. Variable importance measures for each motif at each time points is normalized by dividing the sum of variable importance measures for all motifs in that specific time point.

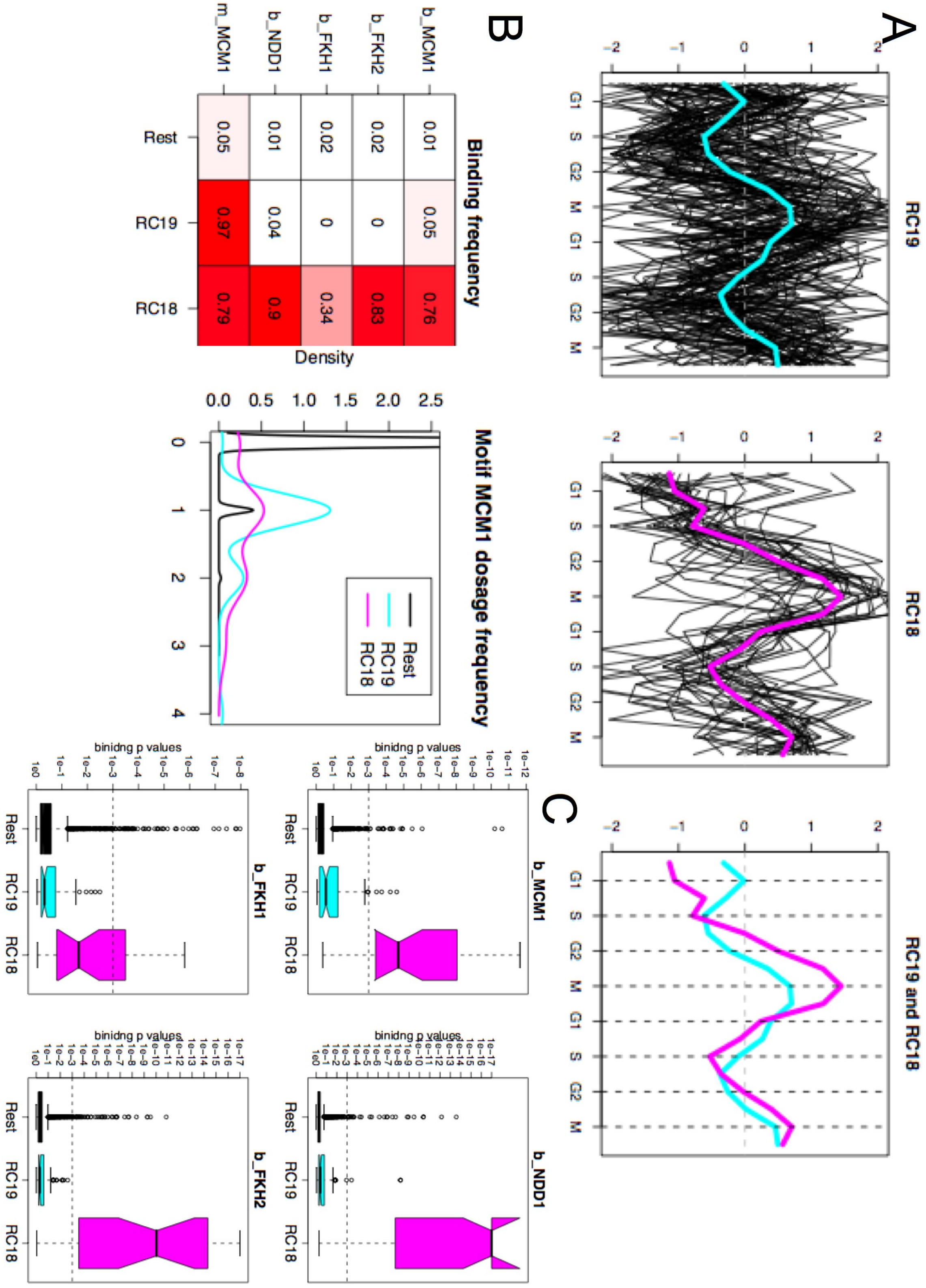

Figure 5: Comparisons of RC18 and RC19 uncovered in yeast cell cycle data by Cho *et al.* using both motifs and TF-binding as predictors. A) Expression profiles of constituent genes. B) Left: binding and motif frequency of feature regulons in the two RCs; Right: MCM1 motif dosages in the two RCs. C) Boxplots of binding p-values of the four binding TFs comparing RC18, RC19 and the rest of the genes.

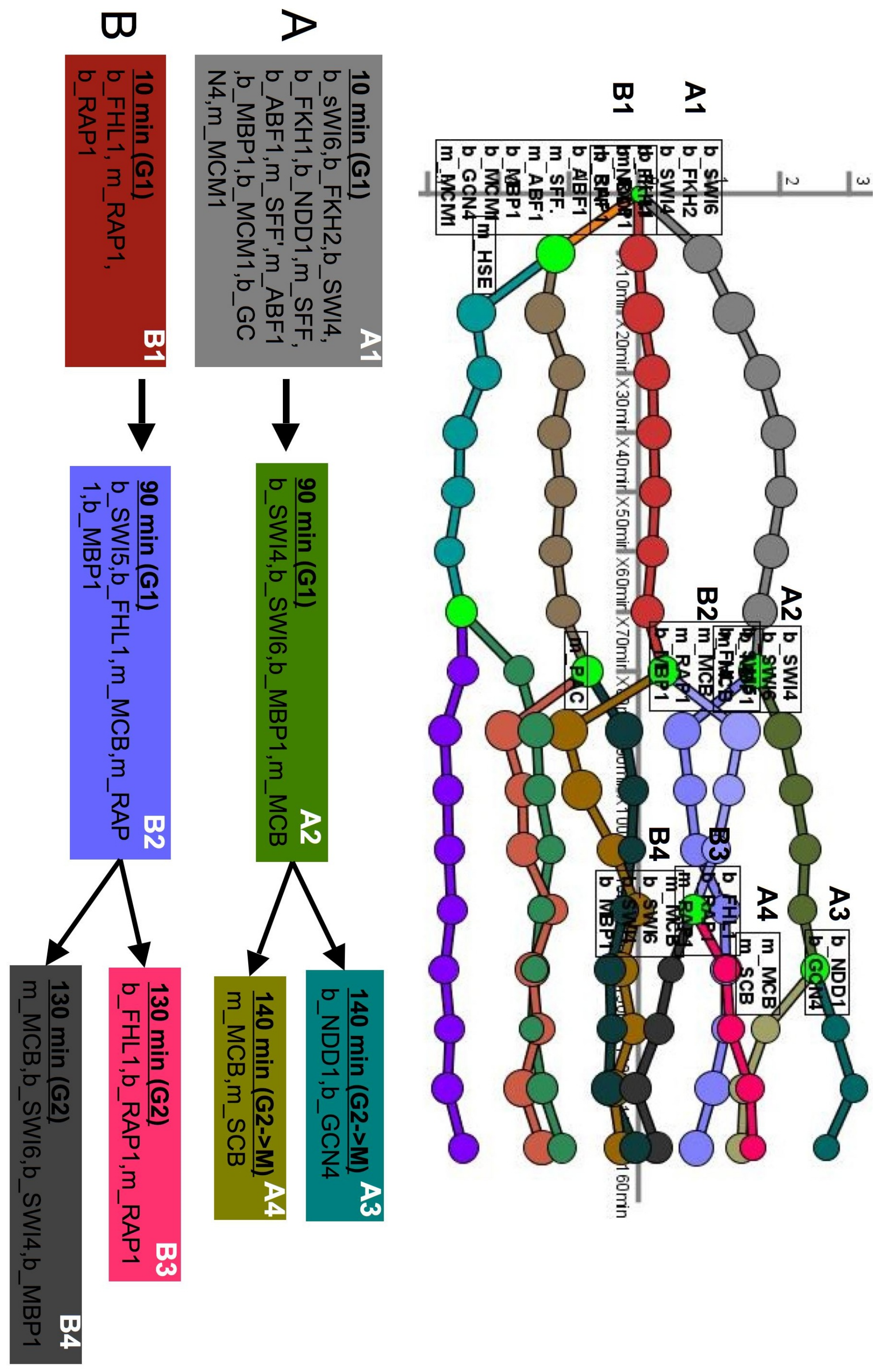

Figure 6: Dynamic regulatory map for yeast response to cell cycle (Cho *et al.*) derived using DREM (*http://www.sb.cs.cmu.edu/drem*). To facilitate interpretation, labeled TFs associated with their respective nodes are presented in a flow chart format in the lower panel. TF boxes from upper and lower panels were matched by their numbers A1-A4 and B1-B4.

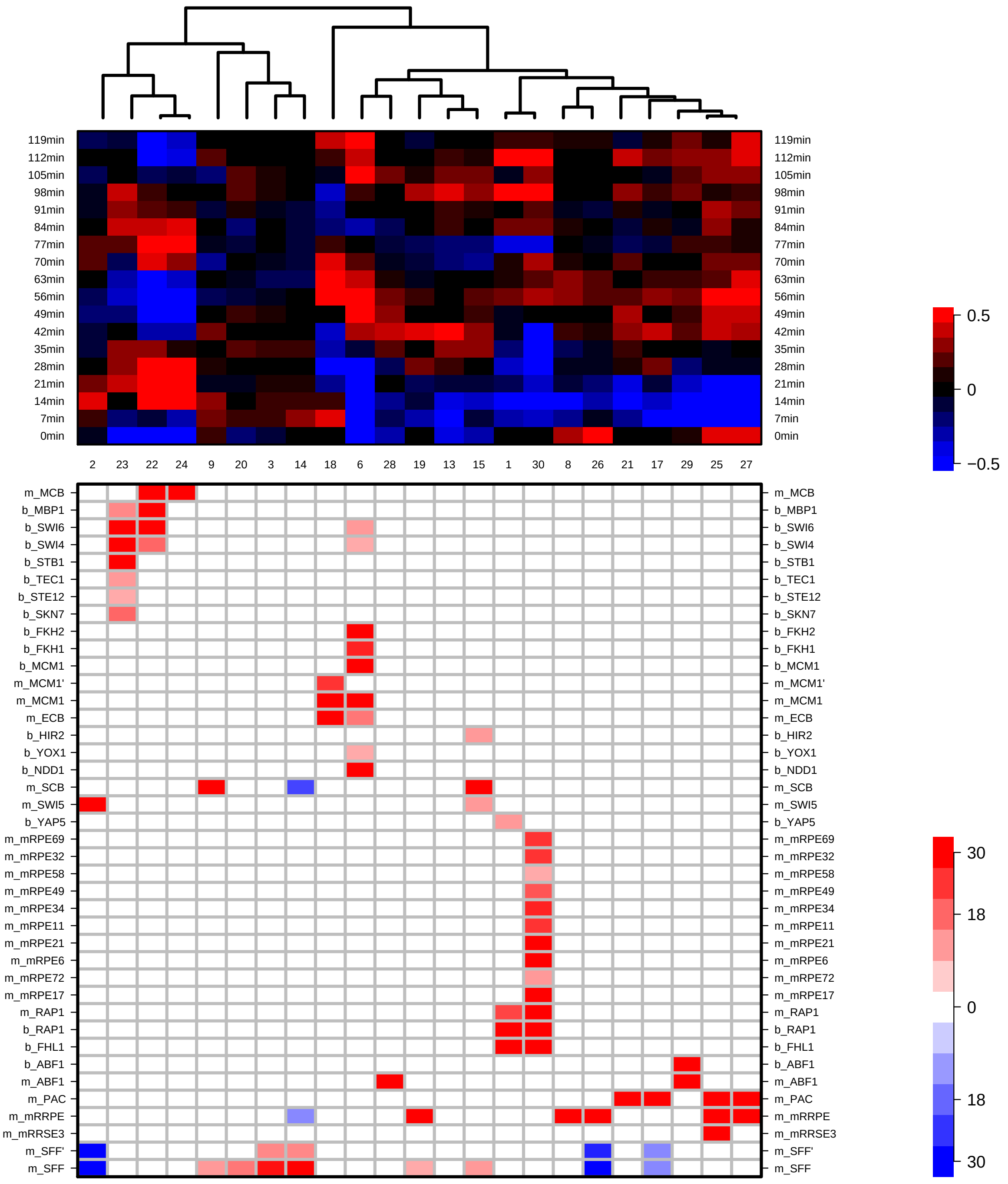

Figure 7: RC diagram of the cell cycle data by Spellman *et al.* using both motifs and TF-binding as predictors. The top section of the graph shows the average expression profile of the genes in a specific RC, which is clustered based on Pearson correlation and average linkage. The bottom section depicts signature regulons in the corresponding RC. Motif regulons have the "m_" prefix whereas TF-binding regulons have the "b_" prefix. The color red indicates enrichment $-log_{10}p$ values by a Chi-square test of association; the color blue corresponds to the depletion $-log_{10}p$ values.

% in common		Cho *et al.* RC6 (MCB)	Cho *et al.* RC20 (Mbp1,Swi4, Swi6,Stb1)	Cho *et al.* RC19 (MCM1)	Cho *et al.* RC18 (MCM1, Fkh1,Fkh2,Ndd1,Mcm1)	Cho *et al.* RC16 (RAP1,RPE,Rap1,Fhl1)
Spellman *et al.*	RC24 (MCB)	94	0	0	0	0
Spellman *et al.*	RC22/23 (Mbp1,Swi4, Swi6)	1	82	0	4	4
Spellman *et al.*	RC18 (MCM1)	0	0	71	11	0
Spellman *et al.*	RC6 (MCM1, Fkh1,Fkh2,Ndd1,Mcm1)	0	0	0	71	0
Spellman *et al.*	RC1/30 (RAP1,RPE, Rap1,Fhl1)	0	0	0	0	82

Figure 8: Tabulation of percentage of common genes between select RCs derived from two independent cell cycle data (Spellman *et al.* and Cho *et al*).

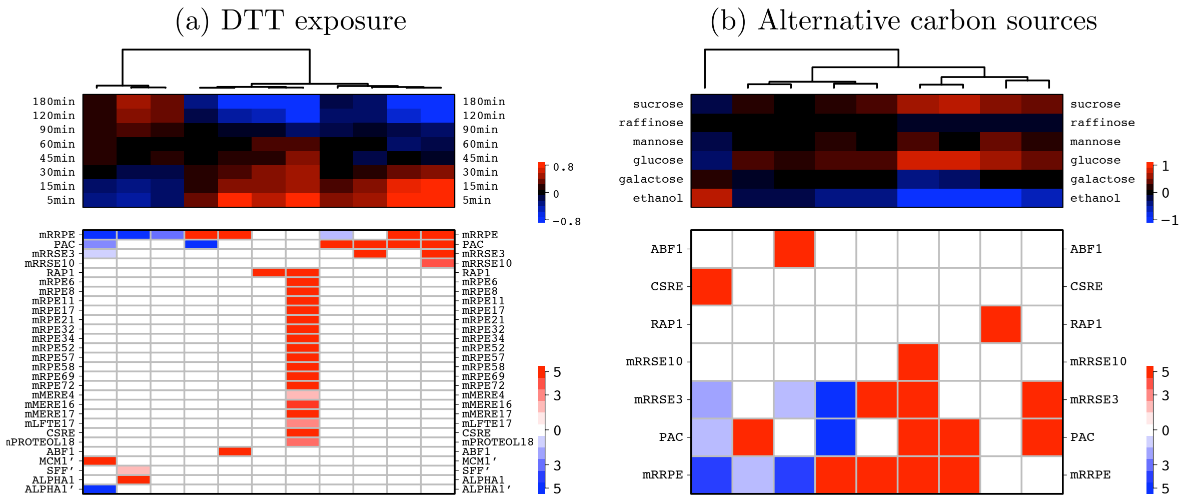

Figure 9: RC diagrams for (a) DTT exposure and (b) alternative carbon sources. The top section shows that dendrogram of hierarchical clustering of the average expression profiles within each RC based on Pearson correlation and average linkage.The bottom section depicts signature motifs in the corresponding RC. The color red indicates enrichment $-log_{10}p-$values by a Chi-square test of association; the color blue corresponds to the depletion $-log_{10}p-$values. The color bar at the lower right hand side is in $-log_{10}p-$ scale and the color signals the direction of the test

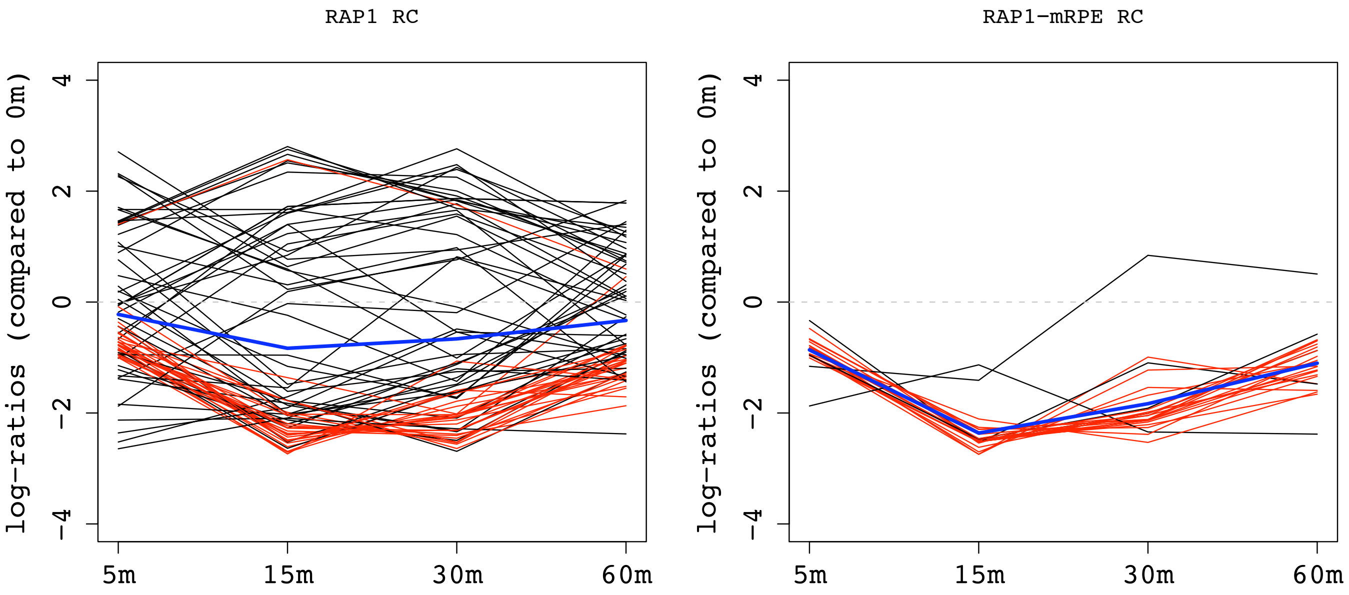

Figure 10: Expression traces of target genes in RAP1 RC (left) and RAP1-mRPE RC (right) of the heat shock data. Traces in red are ribosomal protein genes. The blue lines are average RC expression profiles.
